# Glutathione deficiency in the pathogenesis of SARS-CoV-2 infection and its effects upon the host immune response in severe COVID-19 disease

**DOI:** 10.3389/fmicb.2022.979719

**Published:** 2022-10-06

**Authors:** Carlos A. Labarrere, Ghassan S. Kassab

**Affiliations:** California Medical Innovations Institute, San Diego, CA, United States

**Keywords:** Glutathione, SARS-CoV-2, COVID-19, reactive oxygen species, oxidative stress, acute respiratory distress syndrome, atherosclerosis, atherothrombosis

## Abstract

Severe acute respiratory syndrome coronavirus 2 (SARS-CoV-2) that causes coronavirus disease 19 (COVID-19) has numerous risk factors leading to severe disease with high mortality rate. Oxidative stress with excessive production of reactive oxygen species (ROS) that lower glutathione (GSH) levels seems to be a common pathway associated with the high COVID-19 mortality. GSH is a unique small but powerful molecule paramount for life. It sustains adequate redox cell signaling since a physiologic level of oxidative stress is fundamental for controlling life processes *via* redox signaling, but excessive oxidation causes cell and tissue damage. The water-soluble GSH tripeptide (γ-L-glutamyl-L-cysteinyl-glycine) is present in the cytoplasm of all cells. GSH is at 1–10 mM concentrations in all mammalian tissues (highest concentration in liver) as the most abundant non-protein thiol that protects against excessive oxidative stress. Oxidative stress also activates the Kelch-like ECH-associated protein 1 (Keap1)-Nuclear factor erythroid 2-related factor 2 (Nrf2)-antioxidant response element (ARE) redox regulator pathway, releasing Nrf2 to regulate the expression of genes that control antioxidant, inflammatory and immune system responses, facilitating GSH activity. GSH exists in the thiol-reduced and disulfide-oxidized (GSSG) forms. Reduced GSH is the prevailing form accounting for >98% of total GSH. The concentrations of GSH and GSSG and their molar ratio are indicators of the functionality of the cell and its alteration is related to various human pathological processes including COVID-19. Oxidative stress plays a prominent role in SARS-CoV-2 infection following recognition of the viral S-protein by angiotensin converting enzyme-2 receptor and pattern recognition receptors like toll-like receptors 2 and 4, and activation of transcription factors like nuclear factor kappa B, that subsequently activate nicotinamide adenine dinucleotide phosphate (NADPH) oxidase (NOX) expression succeeded by ROS production. GSH depletion may have a fundamental role in COVID-19 pathophysiology, host immune response and disease severity and mortality. Therapies enhancing GSH could become a cornerstone to reduce severity and fatal outcomes of COVID-19 disease and increasing GSH levels may prevent and subdue the disease. The life value of GSH makes for a paramount research field in biology and medicine and may be key against SARS-CoV-2 infection and COVID-19 disease.

## Introduction

The coronavirus disease 2019 (COVID-19) pandemic affected more than 602.8 million cases with more than 6.4 million deaths reported globally (The New York Times, 2022). Several risk factors including age, hypertension, ischemic heart disease, diabetes, and chronic respiratory disease (Khanfar and Al Qaroot, 2020) increase the fatality rate (Ruan et al., 2020; O’Driscoll et al., 2021) which is directly related with the cytokine storm (Fajgenbaum and June, 2020; Mehta et al., 2020) that can cause acute respiratory distress syndrome, lung injury, respiratory insufficiency, endothelial cell dysfunction, thrombosis, cardiovascular disease and end-organ damage (Klok et al., 2020; Teuwen et al., 2020; Yang L. et al., 2020; Chang et al., 2021; Kaklamanos et al., 2021; Stenmark et al., 2021). All these risk factors have a common characteristic, they are associated with a continuous state of oxidative stress and inflammation, excessive production of free radicals (reactive oxygen and nitrogen species) and endothelial cell dysfunction leading to cardiovascular disease and respiratory failure.

A common pathway affecting all these risk factors involves a low measure of reduced glutathione (i.e., GSH) level (Khanfar and Al Qaroot, 2020; Polonikov, 2020; Silvagno et al., 2020). SARS-CoV-2-infected patients with COVID-19 disease show alterations in the glucose–insulin axis leading to hyperglycemia, hyperinsulinemia and insulin resistance, increased oxidative/nitrosative stress, and significantly decreased vitamin D, thiols, total-antioxidant-capacity, GSH and selenium (Soto et al., 2022). In-depth knowledge of the pathophysiology causing COVID-19-mediated GSH depletion, tissue damage, and acute respiratory distress syndrome is urgently needed. Moreover, the way GSH depletion can lead to immune system failure and make the end organs in danger of oxidative stress-mediated damage needs to be explained. The disturbed redox homeostasis leading to accumulation of reactive oxygen species (ROS) is a common feature in all conditions associated with COVID-19 (Miripour et al., 2020; Pérez de la Lastra et al., 2021). Interestingly, all patients with severe COVID-19 disease and high mortality risk have low basal GSH levels (Khanfar and Al Qaroot, 2020) that could explain an ominous outcome. Several studies pointed out that GSH and the enzymes associated with the GSH pathway are involved in SARS-CoV-2 infection and COVID-19 disease. Recent studies demonstrated that individuals with glutathione transferase omega polymorphisms, genotype variants GSTO1*AA (rs4925) and GSTO2*GG (rs156697), showed significant propensity towards development of clinical manifestations in COVID-19 supporting the significance of these enzymes in the regulation of redox homeostasis and immune response, especially NACHT, LRR, and PYD domains-containing protein 3 (NLRP3) inflammasome activation (Zhao and Zhao, 2020; Djukic et al., 2022). Combined glutathione S-transferase (GST)P1 (rs1138272 and rs1695) and GSTM3 genotypes showed cumulative risk regarding both occurrence and severity of COVID-19 (Coric et al., 2021). COVID-19 patients with the GSTT1-null genotype experience higher mortality (Saadat, 2020; Abbas et al., 2021). Diabetic COVID-19 patients have high cellular oxidative stress, evidenced by decreased extracellular superoxide dismutase 3 levels (Kumar D.S. et al., 2022). Low glutathione S-transferase P1 levels are associated with higher mortality in COVID-19 patients, and high levels of glutathione S-transferase P1 possibly offer protection for cellular redox reactions in severe COVID-19 infection, preventing deterioration especially in patients with co-morbidities like diabetes (Kumar D.S. et al., 2022).

Acute respiratory distress syndrome (ARDS) is considered as a decisive cause of death in COVID-19 disease (Gralinski and Baric, 2015; Quan et al., 2021). Cytokine storm and oxidative stress are the main participants in the development of ARDS during respiratory virus infections (Meftahi et al., 2021). SARS-CoV-2 infection generates massive ROS production and the excessive oxidative damage is responsible for cytokine storm, undermined immunity, tissue damage and severe lung disease causing ARDS and death (Silvagno et al., 2020). Therefore, augmenting tissue GSH levels may lower COVID-19 severity and mortality rates.

Old age, a consequence of aging, is one of the most important risk factors for SARS-CoV-2 infection and development of COVID-19 disease. Aging is directly related to the damage caused by free radicals, particularly ROS causing oxidative stress (Sies, 2015; Baş, 2018). Endothelial cells play a fundamental role in chronic oxidative stress and the development of atherosclerosis, thrombosis and lung injury, principal complications of SARS-CoV-2-mediated tissue and organ dysfunction (Chang et al., 2021). Excess mitochondrial ROS production, causes cellular oxidative stress, sustains mitochondrial dysfunction and ROS production and perpetuates inflammation (Chang et al., 2021). SARS-CoV-2 can activate mitochondrial ROS production, especially in older individuals having ROS overproduction, enhancing oxidative stress and promoting endothelial dysfunction, cardiovascular disease and lung injury. The ROS-mediated damage is further enhanced in older individuals since during aging, GSH levels appear to diminish in numerous tissues, thereby placing cells at increased risk of stress-related death (Maher, 2005). Low GSH in aging is associated with lower intake of GSH precursors, mainly cysteine, and lower GSH synthesis evidencing diminished function of nuclear factor erythroid 2–related factor 2 (Nrf2)-dependent inductive mechanisms that enhance glutamate cysteine ligase expression, rate limiting factor for the synthesis of GSH (McCarty and DiNicolantonio, 2015).

**Figure 1:**
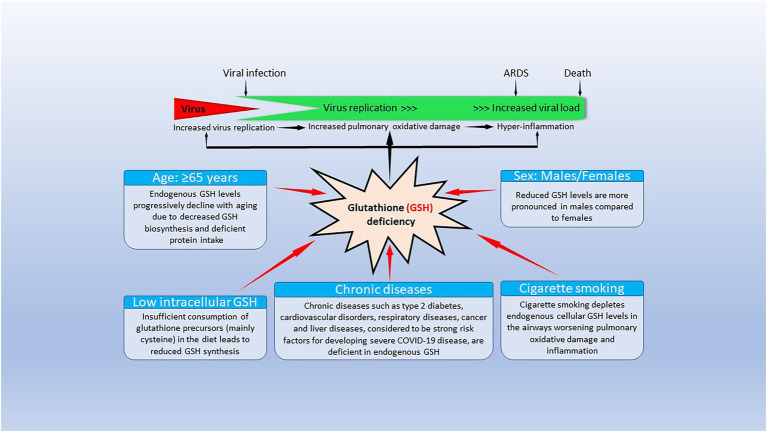
Factors causing endogenous glutathione (GSH) deficiency and GSH deficiency-mediated mechanisms contributing to coronavirus disease 19 (COVID-19) pathogenesis and outcomes. The bottom part of the figure shows that risk factors for severe COVID-19 infection lead to decrease/depletion of intracellular GSH. The top part of the figure shows potential GSH deficiency-mediated mechanisms that could influence clinical manifestations and outcomes in COVID-19 disease. Modified from Polonikov (2020).

All COVID-19 risk factors are associated with reduced GSH levels (Figure 1). As noted previously, increasing age is linked to reduced GSH levels (Dröge, 2002a,b), which can be the result of extensive GSH oxidation and/or reduced pool of cell thiols, and cysteine (whey protein) administration enhances GSH levels and increases longevity (Bounous et al., 1989; Dröge, 2002a,b; Andriollo-Sanchez et al., 2005). Hypertension, ischemic heart disease, patients with atherosclerosis and coronary artery disease, diabetes, chronic lung diseases, smoking and obesity are associated with low baseline GSH levels and reduced GSH/oxidized glutathione (GSSG) ratios (Khanfar and Al Qaroot, 2020; Polonikov, 2020). We will discuss the life-sustaining importance of GSH, its relationship with oxidative stress, as well as its synthesis and catabolism, its biological functions and the paramount relevance of GSH in the immune system (especially the innate immune system), in reducing COVID-19 severity and mortality, and the antiviral capabilities of GSH to reduce SARS-CoV-2 infectivity and multiorgan failure secondary to a cytokine storm in COVID-19 disease.

### Oxidative stress and antioxidants in SARS-CoV-2

Small increase in ROS cellular levels act as signaling molecules in the preservation of cell’s physiological functions — a process known as redox biology; while excessively high levels of ROS causing lipid, protein and DNA cell damage are known as oxidative stress (Schieber and Chandel, 2014). Oxidative stress is a fundamental concept in biology introduced by the first time by Sies (2015). “Oxidative stress is an imbalance between oxidants and antioxidants in favor of the oxidants, leading to a disruption of redox signaling and control and/or molecular damage” (Sies, 2015) that harm lipids, proteins and DNA (Schieber and Chandel, 2014). The prooxidant oxidative stress imbalance needs an antioxidant system able to balance it and the principal role in antioxidant defense is carried out by antioxidant enzymes, with the paramount involvement of small-molecule antioxidant compounds like GSH. Oxidative stress responses clarified the functioning of central principal switches like nuclear factor (NF)-κB (NFκB) or the Kelch-like ECH-associated protein 1 (Keap1)-Nuclear factor erythroid 2-related factor2 (Nrf2)-antioxidant response element (ARE) redox regulator pathway (Sies, 2015; Yamamoto et al., 2018; Cuadrado et al., 2019; Herengt et al., 2021). Under oxidative stress and induced by excessive ROS generation, Nrf2 is released from its inhibitor Keap1, allowing its translocation into the nucleus (Kasprzak et al., 2020), where it binds to the antioxidant response element (ARE) present in the DNA sequence of numerous antioxidant enzymes like glutathione-S-transferase, γ-glutamyl cysteine synthetase (glutamate cysteine ligase), heme oxygenase 1, and paraoxonase-1 inducing their transcription, managing the phase II response to oxidative stress (Kasprzak et al., 2020). Nrf2 also regulates the expression of genes that control inflammatory and immune system responses (Kasprzak et al., 2020). The presence of Nrf2 and its inhibitor Keap1 in plasma is associated with damaged vascular endothelial cell-, macrophage-and other cell-associated leakage secondary to the loss of cell membrane integrity due to lipid peroxidation following chronic inflammation and oxidative stress (Kasprzak et al., 2020), and Nrf2 and Keap1 in circulation are markers of severe inflammation and oxidative stress (Tu et al., 2019). Continuous oxidative stress can lead to chronic inflammation, intense cytokine release and a cytokine storm as seen in SARS-CoV-2 infection in COVID-19 disease, and the viral infection enhances oxidative stress creating a fatal vicious circle between oxidative stress and cytokine storm during COVID-19 infection (Delgado-Roche and Mesta, 2020; Meftahi et al., 2021).

Oxidative stress plays a prominent role in innate immunity being closely involved in SARS-CoV-2 infection (Kozlov et al., 2021). The role of oxidative stress in the COVID-19 disease may involve recognition of the viral S-protein by angiotensin converting enzyme-2 (ACE2) receptor and pattern recognition receptors like toll-like receptors 2 and 4, and activation of transcription factors like nuclear factor kappa B, that subsequently activate nicotinamide adenine dinucleotide phosphate (NADPH) oxidase (NOX) expression succeeded by ROS production (Kozlov et al., 2021). Interestingly, excessive ROS production and oxidative stress raises the binding affinity of the spike protein for the human ACE2 receptor (Hati and Bhattacharyya, 2020; Fossum et al., 2022), suggesting that restoring GSH levels would reduce viral entry and SARS-CoV-2 cellular infection. Thus, excessive production of ROS mediates hyper-inflammation and generation of cytokine storm that directly determine both ARDS development and ARDS course severity. ROS are a necessary defense system to combat microbial respiratory infections (Lambeth, 2004), but oxidative stress and the excessive production of ROS by numerous cells including monocytes and macrophages, neutrophils, as well as pulmonary endothelial and epithelial cells play a major role in the development of ARDS and its complications during COVID-19 infections (Meftahi et al., 2021). The high neutrophil to lymphocyte ratio in critically ill patients, the intense neutrophil infiltration in pulmonary capillaries and into pulmonary alveoli, and the increased levels of circulating neutrophil extracellular traps (NETs) clearly show neutrophil involvement/activation favoring intense ROS production and oxidative damage caused by lipid peroxidation, and protein and DNA oxidation (Komaravelli and Casola, 2014; Laforge et al., 2020; Veras et al., 2020; Ng et al., 2021). SARS-CoV-2-mediated NET release can promote lung epithelial cell death unravelling a detrimental role of NETs in the pathophysiology of COVID-19 disease (Veras et al., 2020; Ouwendijk et al., 2021). Extensive persistent inflammation even when SARS-CoV-2-infected cells are only sporadically present at late stages of COVID-19 (Schurink et al., 2020) could also justify low GSH levels in association with over-abundance of cellular ROS, cellular oxidative damage, increased inflammation and cell death pathways activation seen in inflammaging (Zuo et al., 2019; Bharath and Nikolajczyk, 2020; Cunha et al., 2020). Neutrophilia causes excessive ROS production that aggravates the host immunopathological response, leading to a more severe disease (Laforge et al., 2020). In addition to the neutrophil infiltration and ROS release, viral infections decrease antioxidant defenses. They inhibit Nrf2 translocation into the nucleus and enhance NFκB activation promoting inflammation and oxidative damage (Laforge et al., 2020). Nrf2 is the principal transcription factor in charge of protecting cells from oxidative stress through the regulation of cytoprotective genes, including the antioxidant GSH pathway, that controls GSH homeostasis by affecting *de novo* synthesis. It has been shown that Nrf2 modulates the GSH redox state *via* glutathione reductase regulation. Overall, Nrf2 is fundamental for the sustenance of the GSH redox state through glutathione reductase transcriptional regulation and for cell protection against oxidative stress (Harvey et al., 2009).

The overwhelming dominance of ROS generated by enzymes like NADPH oxidases and xanthine oxidase over antioxidants like superoxide dismutase causes cell injury and tissue damage through direct injury, lipid peroxidation and protein oxidation leading to protease release and antioxidant and antiprotease enzyme inactivation as well as alteration of transcription factors activator protein-1 and NFκB. Exposure to pro-oxidant stimuli usually induces Nrf2 activation and upregulation of antioxidant enzyme expression through binding to the antioxidant response element (ARE), localized in the antioxidant enzyme gene promoters; while respiratory viral infections cause antioxidant enzyme expression/activity inhibition associated with reduced Nrf2 nuclear localization, decreased cellular levels and reduced ARE-dependent gene transcription (Komaravelli and Casola, 2014). All these changes lead to cytokine storm characterized by increased expression and release of proinflammatory cytokines that participate in the pathogenesis of ARDS during virus respiratory infections like COVID-19. Proinflammatory cytokines further stimulate ROS overproduction aggravating ARDS and lung damage causing a vicious circle between oxidative stress and cytokine storm. In response to a viral infection, activated cells have enhanced production of the NOX family of NADPH oxidases (Brandes et al., 2014; Panday et al., 2015). NOX family members normally regulate cellular physiological functions, but under abnormal circumstances contribute to the pathogenesis of cell/tissue damage associated with infections and vascular disorders (Panday et al., 2015). The presence of oxidative stress markers like lipid peroxidation, neutrophil reverse trans-endothelial migration (rTEM) and high neutrophil to lymphocyte ratio in patients with COVID-19, facilitates identification of high-risk individuals early in the course of the disease preventing their sudden deterioration (Laforge et al., 2020). Furthermore, increased ACE2 expression in alveolar type II pneumocytes and alveolar macrophages of individuals with severe SARS-CoV-2 disease (ARDS with diffuse alveolar damage) requiring mechanical ventilation (Baker et al., 2021) favors a concomitant increase in oxidative stress in those individuals.

## Glutathione and immune system enhancement

Glutathione is fundamental to sustain an adequate function of the immune system, particularly affecting the lymphocyte activity since low GSH levels inhibit T-cell proliferation and immune response (Dröge and Breitkreutz, 2000; Ghezzi, 2011; Moro-García et al., 2018; Kelly and Pearce, 2020; Khanfar and Al Qaroot, 2020; Shyer et al., 2020). GSH depletion is strongly associated with impaired immune function and with disease development including viral diseases, cancer, cardiovascular diseases, arthritis and diabetes (Sinha et al., 2018; Sharifi-Rad et al., 2020; Silvagno et al., 2020; Fraternale et al., 2021; Matuz-Mares et al., 2021). GSH is essential for immunomodulation of both innate and adaptive immune system functions, including T-lymphocyte proliferation, polymorphonuclear neutrophil phagocytosis, and dendritic cell functions, and is also important for fine-tuning the innate immune response to infection and for the first step of adaptive immunity involving antigen-presenting cell (macrophages, dendritic cells)-related antigen presentation (Morris et al., 2013; Diotallevi et al., 2017). GSH works to modulate the behavior of many immune cells, augmenting both, innate immunity (and trained innate immunity or innate immune memory; Netea et al., 2020; Chumakov et al., 2021; Ferreira et al., 2021; Gong et al., 2021; Brueggeman et al., 2022), severely affected by SARS-CoV-2 viral infection (Polonikov, 2020; Rodrigues et al., 2020; Forcados et al., 2021; Kozlov et al., 2021; Bellanti et al., 2022; Paludan and Mogensen, 2022), and adaptive immunity (Dröge et al., 1991; Dröge and Breitkreutz, 2000; Dröge, 2002c; Ghezzi, 2011; Morris et al., 2013; Fraternale et al., 2017), as well as conferring protection against oxidative stress caused by microbial, parasitic and viral infections such as SARS-CoV-2 that causes COVID-19 disease (Morris et al., 2013; Diotallevi et al., 2017; Derouiche, 2020; Polonikov, 2020; Silvagno et al., 2020; Suhail et al., 2020; Forcados et al., 2021; Pérez de la Lastra et al., 2021; Bellanti et al., 2022; Kumar P. et al., 2022). Persistent and uncontrolled oxidative stress and exacerbating NLRP3 (NOD-, LRR-, and pyrin domain-containing protein 3) inflammasome activation during severe COVID-19 disease (Lage et al., 2022), induce production of pro-inflammatory cytokines, such as IL-1β and IL-18, that can be explained because of sharply decreased macrophage GSH intracellular levels associated with increased GSH efflux (Zhang T. et al., 2021).

Many antioxidant molecules, such as GSH and N-acetylcysteine (NAC), were found to inhibit viral replication through different mechanisms of action (Fraternale et al., 2006). GSH levels in macrophages, directly affect the Th1/Th2 cytokine response, and more specifically, GSH depletion inhibits Th1-associated cytokine production and/or promotes Th2 associated responses (Fraternale et al., 2006). Cell-mediated immunity primarily needs protein antigen degradation in the endocytic vesicles of antigen presenting cells (macrophages, dendritic cells), to be able to present smaller peptides on the cell surface through major histocompatibility complex antigens to activate antigen-specific T cell proliferation. One of the initial steps in antigen degradation and processing is the reduction of disulfide bonds, that requires GSH; and although GSH inhibits production of most inflammatory cytokines, it is needed to keep an adequate interferon gamma production by dendritic cells, essential for intracellular pathogen host defense (Ghezzi, 2011; Lee and Ashkar, 2018; Calder, 2020; Fraternale et al., 2021). The principal function of endogenous GSH is not to limit inflammation but to fine-tune the innate immune response to infection (Diotallevi et al., 2017; De Flora et al., 2020; Silvagno et al., 2020; Ferreira et al., 2021). GSH is capable of scavenging ROS through Nrf2-mediated heme oxygenase-1 induction and enhancing M1-like macrophage polarization regulation, showing that GSH may be a useful strategy to increase the human defense system (Mittal et al., 2014; Kwon et al., 2019; Funes et al., 2020). Strategies to enhance intracellular GSH levels such as supplementation of additional sources of cysteine (Deneke and Fanburg, 1989; Dröge et al., 1991; Lands et al., 1999; Dröge and Breitkreutz, 2000; Ghezzi et al., 2019; Gould and Pazdro, 2019; Minich and Brown, 2019; Castejon et al., 2021), oral and intravenous GSH (Cazzola et al., 2021), and sublingual and/or oral liposomal GSH administration (Schmitt et al., 2015; Campolo et al., 2017; Sinha et al., 2018; Guloyan et al., 2020; To et al., 2021) will also help to improve the immunological functions. The GSH and NAC digestive degradation occurring during oral treatments lead to consider GSH and NAC nebulization as a viable alternative to manage early stages of COVID-19 disease (Santos Duarte Lana et al., 2021).

GSH increases activation of cytotoxic T cells *in vivo*, and adequate functioning of T lymphocytes and other cells depends upon *cellular supplies of cysteine* (Edinger and Thompson, 2002; Garg et al., 2011; Levring et al., 2015). Cells acquire cysteine mainly by macrophage and lymphocyte uptake, and impaired immune responses are associated with a reduction in GSH concentration (Dröge and Breitkreutz, 2000; Edinger and Thompson, 2002; Garg et al., 2011; Ghezzi, 2011; Diotallevi et al., 2017; Calder, 2020). GSH depletion triggers the lymphocyte’s apoptotic cascade leading to lymphopenia that affects for the most part T lymphocytes, and lymphopenia is directly associated with severe disease and high mortality rate in COVID-19 patients (Pallardó et al., 2009; Circu and Aw, 2012; Huang and Pranata, 2020; Ruan et al., 2020; Wang et al., 2021b; Zaboli et al., 2021). GSH is of paramount importance for the appropriate function of the immune system in general and particularly lymphocytes since low GSH levels inhibit T lymphocytes proliferation and subsequently disturbs the immune response (Hamilos et al., 1989; Dröge and Breitkreutz, 2000; Hadzic et al., 2005; Kesarwani et al., 2013; Fraternale et al., 2017; Moro-García et al., 2018; Khanfar and Al Qaroot, 2020). The decreased immune response could be reversed by the administration of N-acetylcysteine (Atkuri et al., 2007; Rushworth and Megson, 2014; De Flora et al., 2020; Bourgonje et al., 2021; Schwalfenberg, 2021) which elevates tissue GSH levels by providing the amino acid cysteine (Atkuri et al., 2007; Rushworth and Megson, 2014; De Flora et al., 2020; Bourgonje et al., 2021; Schwalfenberg, 2021). Low GSH levels inhibit interleukin-2 production, which induces lymphocyte proliferation (Chang et al., 1999; Hadzic et al., 2005).

T-cell function can be recuperated following administration of GSH precursors like N-acetyl cysteine and cysteine (Dröge and Breitkreutz, 2000; Ghezzi, 2011; Aquilano et al., 2014; De Flora et al., 2020; Guloyan et al., 2020; Pedre et al., 2021). A deepest effect of low GSH levels on the immune system would be the induction of lymphocytes’ apoptotic cascade. GSH depletion is needed for apoptosis to be triggered in the lymphocytes regardless of ROS (Franco et al., 2007a; Pallardó et al., 2009; Circu and Aw, 2012; Franco and Cidlowski, 2012; Khanfar and Al Qaroot, 2020). In order to induce T lymphocyte apoptosis, GSH must be pumped out of the cells (Franco and Cidlowski, 2006, 2012; Franco et al., 2007a; Ballatori et al., 2009; Khanfar and Al Qaroot, 2020). The GSH effects on apoptosis and inhibition of T-cell proliferation could explain why patients with SARS-CoV-2 infection and COVID-19 disease develop lymphopenia and subsequent failure of the immune system (Khanfar and Al Qaroot, 2020). A way to explain cell death associated with reduced levels of GSH is ferroptosis, a unique iron-dependent form of non-apoptotic cell death, characterized by lipid peroxidation with ROS accumulation due to GSH peroxidase inactivation and high levels of GSH consumption; ferroptosis has been proposed to be involved in COVID-19-related brain injury (Zhang et al., 2022). Immune system failure could lead to uncontrolled replication of the SARS-CoV-2 virus, secondary infections and continuous shedding of the virus in patients who die from COVID-19 regardless of the time passed from the start of the infection (Ruan et al., 2020; Sharma et al., 2020; Zhou et al., 2020; Proal and Van Elzakker, 2021; Trougakos et al., 2021). GSH is essential for the appropriate function of all components of the immune system, particularly T-lymphocytes, macrophages and neutrophils; and the failure of the immune system combined with the loss of GSH’s protective effect as an antioxidant may explain the progression of the disease into acute respiratory distress syndrome/acute lung injury.

## Glutathione and SARS-CoV-2

**Figure 2:**
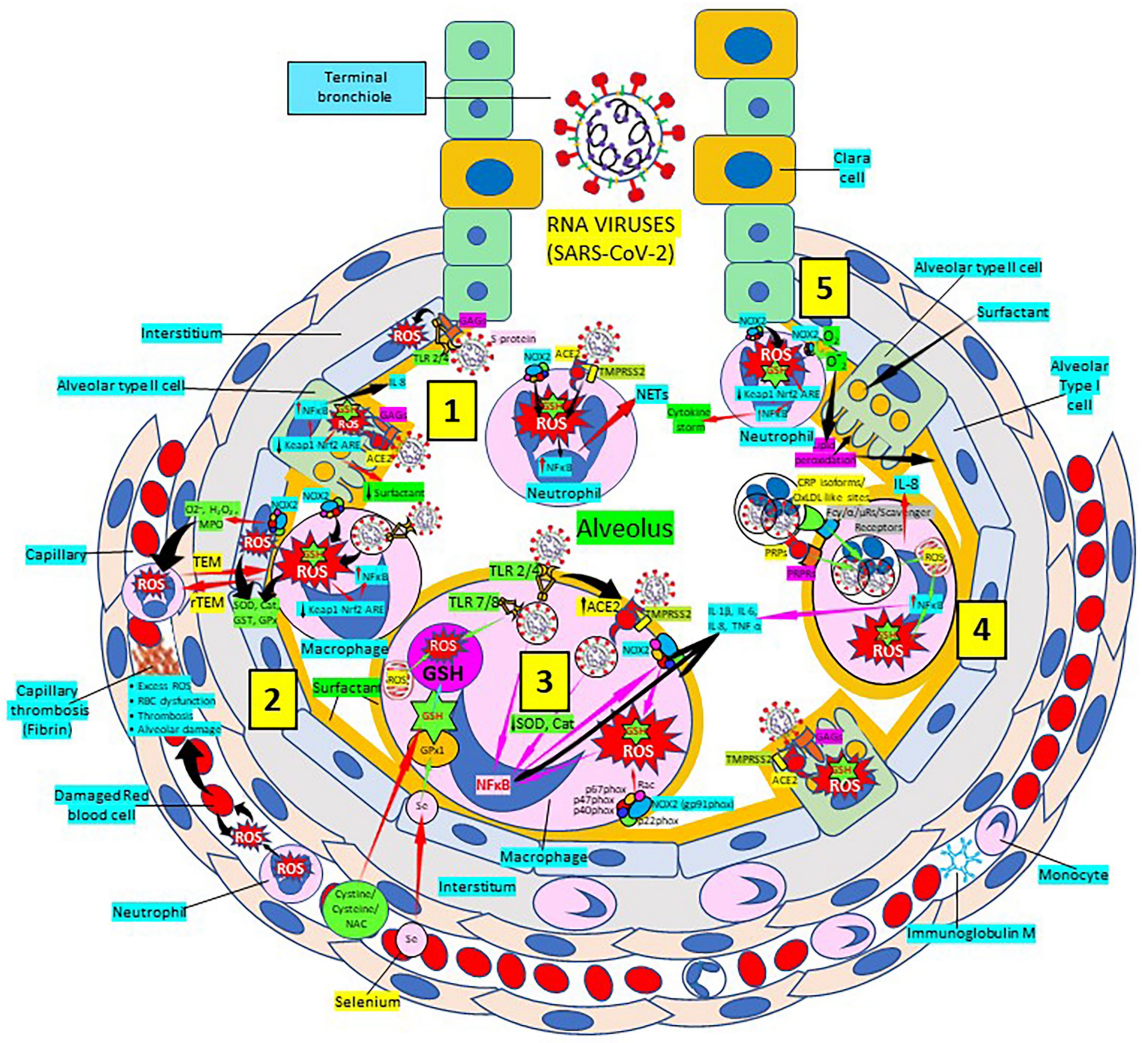
Severe acute respiratory syndrome coronavirus 2 (SARS-CoV-2) pulmonary infection, oxidative stress and antioxidant defenses. [1] After entry of SARS-CoV-2 into the alveolus, viruses invade type II alveolar cells through angiotensin-converting enzyme 2 receptors (ACE2) and glycosaminoglycans (GAGs), and infected cells increase reactive oxygen species (ROS) production, reduce Kelch-like ECH-associated protein 1 (Keap1)-Nuclear factor erythroid 2-related factor 2 (Nrf2)-antioxidant response element (ARE) redox regulator pathway and become defective for surfactant production. Infected cells activate nuclear factor (NF)-κB and release cytokines like interleukin (IL)-8. Alveolar type I cells augment ROS production *via* toll-like receptors (TLRs) 2 and 4. SARS-CoV-2 enhances neutrophil extracellular trap (NET) release and increases ROS production [2] SARS-CoV-2 augments macrophage’s ROS production, inhibiting Nrf2 activation and enhancing NF-κB upregulation. ROS are counterbalanced by enzymes like superoxide dismutase (SOD), catalase (Cat), glutathione S-transferase (GST) and glutathione peroxidase (GPx) to protect cells from oxidative damage caused by nicotinamide adenine-dinucleotide phosphate (NADPH) oxidase 2 (NOX2), superoxide (O_2_^−^), hydrogen peroxide (H_2_O_2_), and myeloperoxidase (MPO). Capillary neutrophils migrate to and from alveoli by trans-endothelial (TEM) and reverse transmigration (rTEM), respectively. SARS-CoV-2 infection can cause excessive ROS production in capillaries, red blood cell (RBC) dysfunction, thrombosis and alveolar damage. [3] SARS-CoV-2-infected macrophages (*via* ACE2 and TLRs) reduce enzymes like SOD and Cat, among others, and activate NF-κB. NOX2 activation increases ROS production that enhance NF-κB activation. Activated alveolar macrophages release increased levels of IL-1β, IL-6, IL-8 and tumor necrosis factor (TNF)-α. Glutathione (GSH) precursors (Cystine, cysteine, N-acetyl cysteine, NAC), and selenium (Se) restore GSH and GPx, respectively, to counteract the effects of ROS. [4] Alveolar macrophages engulf SARS-CoV-2-infected apoptotic cells *via* Fc (γ/α/μ) and scavenger receptors and/or pattern recognition protein receptors (PRPRs) leading to increased ROS production, NFκB activation and cytokine release; and infected alveolar type II cells enhance inflammation. [5] Neutrophils contribute to O_2_^−^ production, lipid peroxidation and increased oxidative stress, Keap-1-Nrf2-ARE signaling pathway reduction and NFκB activation promoting cytokine storm. Abbreviations: TMPRSS2: Transmembrane protease Serine 2; PRPs: pattern recognition proteins.

SARS-CoV-2 infection causes intense inflammation which is associated with damaging systemic events that include excessive ROS production, oxidative stress, ROS-mediated apoptosis/cell death, dysregulation of iron homeostasis, hypercoagulability and thrombus formation (Kernan and Carcillo, 2017; Moore and June, 2020; Phua et al., 2020; Vinciguerra et al., 2020; Zhou et al., 2020; Figure 2). Several viral infections, and the progression of virus-induced diseases, especially those associated with COVID-19, are characterized by an alteration in the intracellular redox balance (Polonikov, 2020). Oxidative stress reflects an imbalance between increased ROS production and reduced cellular antioxidant capabilities. This imbalance disallows reactive intermediate detoxification by the cell biological systems. ROS production and associated inflammation are closely related to aging and numerous chronic diseases as diabetes, cardiovascular and respiratory diseases, known risk factors for developing severe illness and death in patients with SARS-CoV-2 and COVID-19 disease.

**Figure 3:**
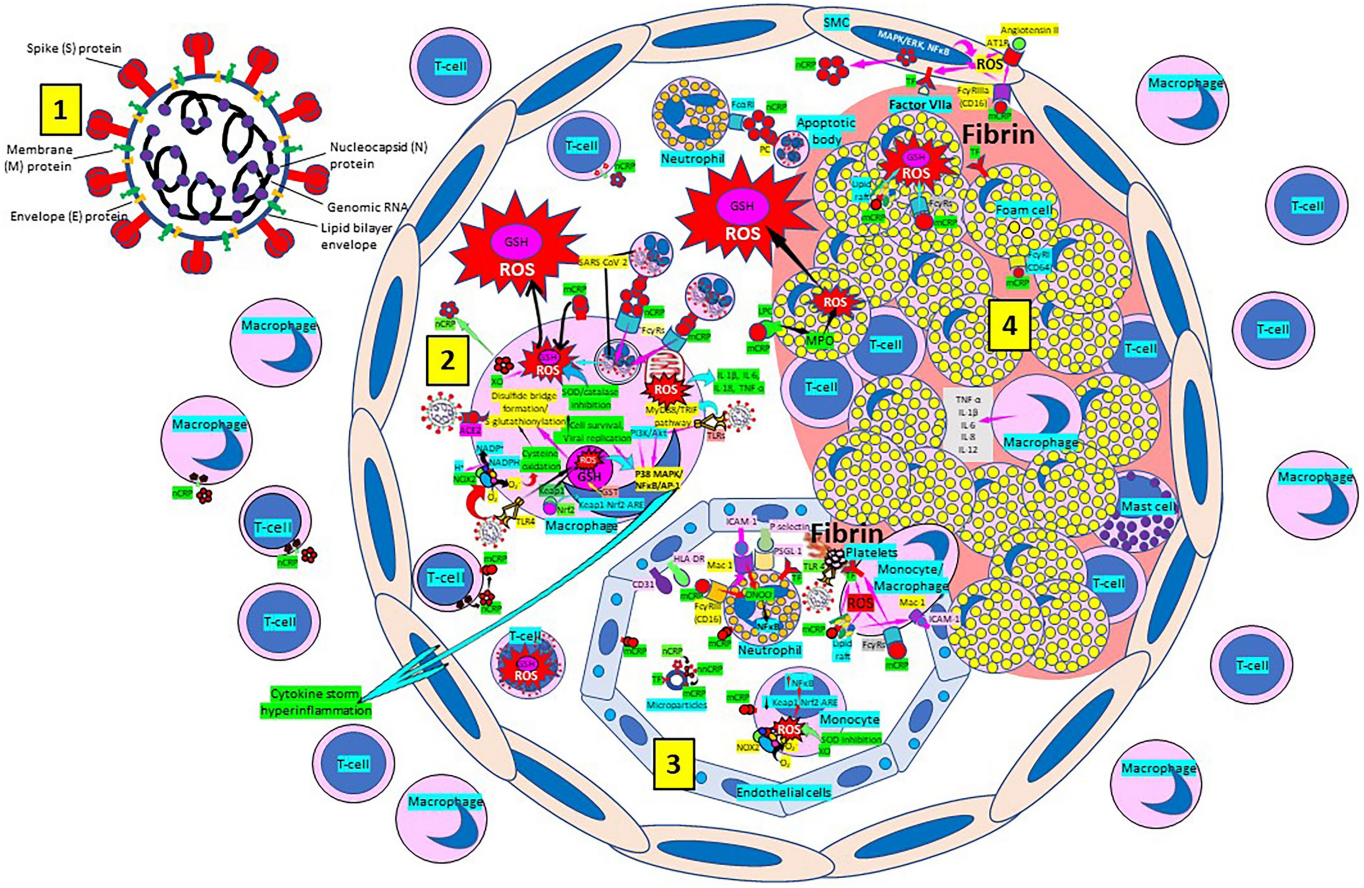
Severe acute respiratory syndrome coronavirus-2 (SARS-CoV-2) enhances oxidative stress and atherosclerosis progression. [1] SARS-CoV-2 structure. [2] SARS-CoV-2 viruses facilitate oxidative stress and inflammation in the arterial intima. Native C-reactive protein (nCRP), a marker of severe SARS-CoV-2 produced in liver, macrophages, lymphocytes, smooth muscle cells (SMC) and other cells, promotes inflammation through monomeric CRP (mCRP) enhancing intimal oxidative stress. SARS-CoV-2 binds macrophage toll-like receptor (TLR) 4 and facilitates nicotinamide adenine dinucleotide phosphate (NADP) H oxidase 2 (Nox2) activity and superoxide (O_2_^−^) production causing cysteine oxidation, disulfide bridge formation and S-glutathionylation. Xanthine oxidase (XO) and inhibition of superoxide dismutase (SOD)/catalase further facilitate O_2_^−^ cellular activity and ROS generation. SARS-CoV-2 can bind TLRs 2 and 4 and activate transcription factors like nuclear factor (NF)-κB facilitating cytokine storm and hyperinflammation. Excessive mitochondrial reactive oxygen species (ROS) generation further enhances cytokine production. CRP (nCRP, mCRP) can facilitate macrophage and neutrophil uptake of SARS-CoV-2-infected apoptotic cells through Fcγ and Fcα receptors, respectively (FcRs). Oxidative stress also activates the Kelch-like ECH-associated protein 1 (Keap1)-Nuclear factor erythroid 2-related factor2 (Nrf2)-antioxidant response element (ARE) redox regulator pathway in monocytes (see [3] and macrophages, releasing Nrf2 to regulate the expression of genes that control antioxidant enzymes like glutathione S-transferase (GST)), facilitating glutathione (GSH) activity. Macrophages, Tlymphocytes, neutrophils and SMCs can generate mCRP increasing inflammation. [3] Monocytes, macrophages, neutrophils, endothelial cells and microparticles can generate mCRP, increase O_2_^−^ and ROS formation and reactive nitrogen species like peroxinitrite (ONOO^−^), and tissue factor (TF) expression enhancing oxidation, inflammation and thrombosis. TLR 4-mediated SARS-CoV-2-binding to platelets promotes thrombosis, mCRP binding to lipid rafts and FcγRs enhances inflammation and endothelial activation allows intimal cell migration. [4] Foam cells and smooth muscle cells associated with atherosclerotic plaques enhance ROS formation, cytokine release and tissue factor (TF)-mediated fibrin deposition. MAPK/ERK, mitogen-activated protein kinase/extracellular signal-regulated kinase; AT1R, Angiotensin II type 1 receptor; PC, phosphorylcholine; LPC, lysophosphatidylcholine; MPO, myeloperoxidase; nnCRP, non-native CRP; TNF, tumor necrosis factor; IL, interleukin; ACE, angiotensin converting enzyme; MyD88/TRIF, myeloid differentiation primary response88/TIR-domain-containing adapter-inducing interferon-β; PI3K/Akt, phosphatidylinositol-3-kinase/protein kinase B; AP-1, activator protein 1; CD31, cluster of differentiation 31; ICAM-1, intercellular adhesion molecule-1; Mac-1, macrophage-1 antigen; PSGL-1, P-selectin glycoprotein ligand-1; HLA-DR, Human Leukocyte Antigen – DR isotype.

Atherosclerosis, a chronic inflammatory disease, may be an ideal environment for the high viral replication capabilities of SARS-CoV-2 in human cells, enhancing hyper-inflammation secondary to immune system dysregulation (Figure 3) that leads to adverse outcomes, as shown in patients with cardiovascular risk factors. In a vicious circle, feeding itself, SARS-CoV-2 may aggravate the evolution of atherosclerosis as a result of excessive and aberrant plasmatic concentration of cytokines (Vinciguerra et al., 2020; Labarrere and Kassab, 2021). Atherosclerosis progression, as a chronic inflammatory mechanism, is characterized by immune system dysregulation associated with increased pro-inflammatory cytokine production, including interleukin 6 (IL-6), tumor necrosis factor-α (TNF-α), and IL-1β (Vinciguerra et al., 2020; Labarrere and Kassab, 2021). C-reactive protein (CRP), an active regulator of host innate immunity, is a biomarker of severe COVID-19 disease, including lung and atherosclerotic disease progression; strongly predicts the need for mechanical ventilation; and may guide intensification of treatment of COVID-19-associated uncontrolled inflammation (Potempa et al., 2020; Labarrere and Kassab, 2021; Luan et al., 2021). Macrophage activation and foam cell formation may explain the elevated CRP serum levels and contribute to disease progression (Figure 3). CRP-mediated inflammation in atherosclerosis during SARS-CoV-2 infection may be explained by the presence of monomeric CRP (mCRP) in the lesions (Potempa et al., 2020; Fazal, 2021; Fendl et al., 2021; Labarrere and Kassab, 2021; Luan et al., 2021; Mosquera-Sulbaran et al., 2021). The affinity of SARS-CoV-2 for ACE2 receptors makes the virus prone to cause vascular infection that could explain atherosclerosis progression and arterial and venous thrombosis (Vinciguerra et al., 2020; Labarrere and Kassab, 2021). Endothelial injury generated directly by intracellular viral replication and by ACE2 downregulation, exposing cells to angiotensin II in the absence of the modulator effects of angiotensin 1–7 (Vinciguerra et al., 2020), and vascular chronic inflammation promoting the development of tissue macrophages overloaded by cholesterol (foam cells), both increase the possibility of acquiring a severe COVID-19 infection (Vinciguerra et al., 2020; Labarrere and Kassab, 2021).

**Figure 4:**
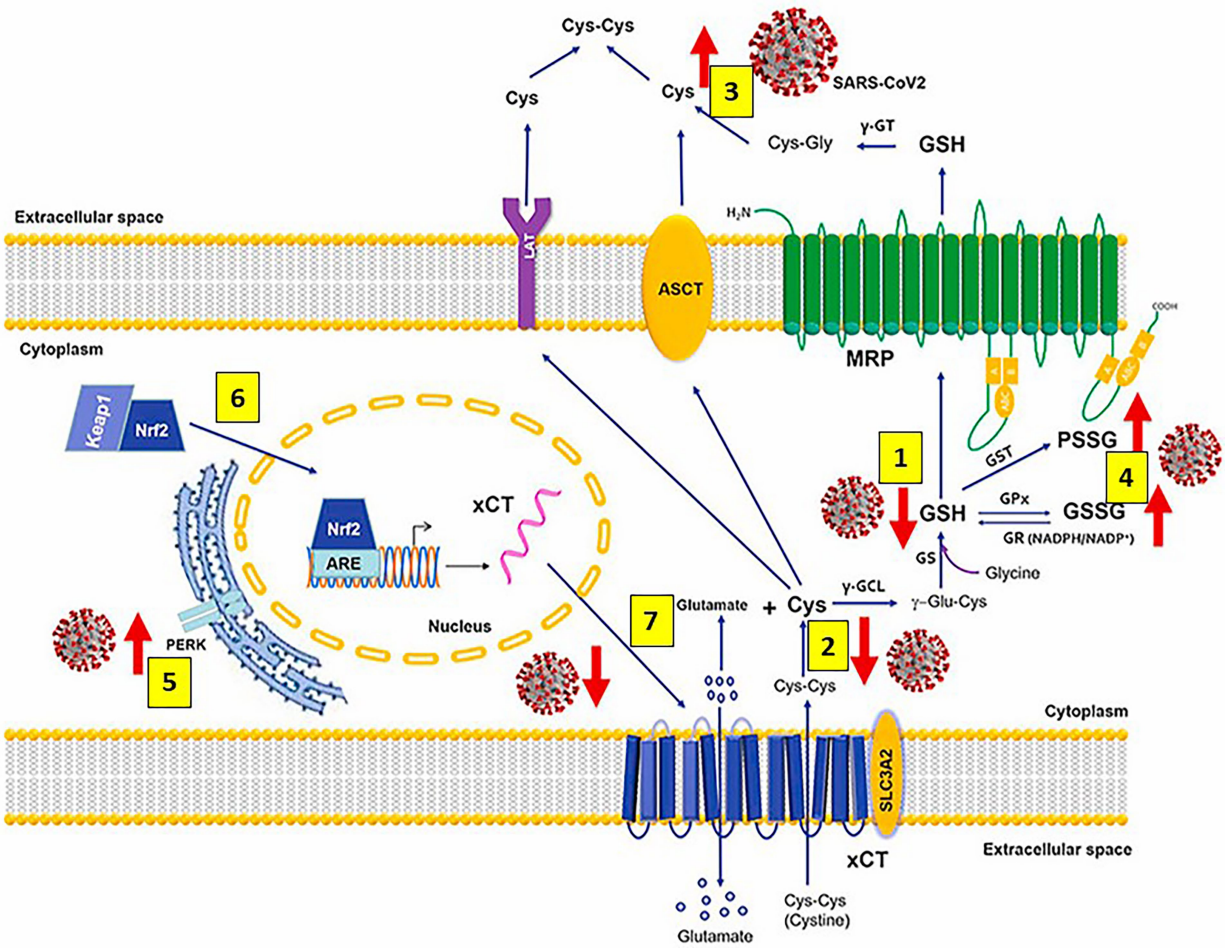
Severe acute respiratory syndrome coronavirus 2 (SARS-CoV-2) infection alters metabolism and redox function of cellular glutathione (GSH). SARS-CoV-2 markedly decreases GSH levels [1], that could be explained by lower intake of the GSH precursor cysteine (Cys) [2] and increased efflux of cellular thiols [3]. Increased levels of oxidized glutathione (GSSG) and protein glutathionylation [4] along with upregulation of endoplasmic reticulum stress marker protein kinase R (PKR)-like endoplasmic reticulum kinase (PERK) [5] are also observed. Antivirals activate the Kelch-like ECH-associated protein 1 (Keap1)-Nuclear factor erythroid 2-related factor2 (Nrf2)-antioxidant response element (ARE) redox regulator pathway, releasing Nrf2 [6] to regulate the expression of genes that control antioxidant, inflammatory and immune system responses (including the cystine (cys-cys)/glutamate transporter xCT and the membrane transporter multidrug resistance protein [MRP], which are decreased and markedly upregulated, respectively, during infection); restoring GSH levels in the infected cells and facilitating GSH synthesis [7] and activity. Abbreviations: γ-GT: γ-glutamyl transferase; ASCT: alanine-serine-cysteine transporter; LAT: L-type amino acid transporter; PSSG: S-glutathionylated Proteins; GST: glutathione-S-transferase; γ-GCL: γ-glutamate cysteine ligase; γ-glutamyl cysteine; GST: glutathione-S-transferase; GPx: glutathione peroxidase; GR: glutathione reductase; NADPH: reduced NADP+; NADP+: Nicotinamide adenine dinucleotide phosphate; GS: glutathione synthetase; xCT (SLC7A11)/SLC3A2: cystine/glutamate transporter light (xCT [SLC7A11]) and heavy (SLC3A2) chains. Modified from Bartolini et al. (2021).

As mentioned previously, GSH, a tripeptide containing glutamate, cysteine and glycine, (L-γ-glutamyl-L-cysteinyl-glycine) is the master and most potent cellular antioxidant and the most abundant low molecular weight thiol that plays a crucial role in antioxidant defense against cellular ROS/reactive nitrogen species (RNS)-mediated oxidative damage and in the regulation of numerous metabolic pathways essential for maintaining whole body homeostasis. GSH synthesis catalyzed sequentially by two cytosolic enzymes, γ-glutamyl-cysteine synthetase (GCS) and GSH synthetase is part of virtually all cell types, and the liver is the major GSH producer and exporter. Preservation of the highest (millimolar) concentrations of reduced GSH in most cell types highlights GSH vital and multifunctional roles in controlling various biological processes like detoxification of foreign and endogenous compounds, protein folding, regeneration of vitamins C and E, maintenance of mitochondrial function, regulation of cell cycle and cell proliferation, apoptosis, immune response, and multiple other cellular and biological functions, particularly important, antiviral defense (Meister and Anderson, 1983; Townsend et al., 2003; Sastre et al., 2005; Franco et al., 2007b; Valko et al., 2007; Forman et al., 2009; Diaz-Vivancos et al., 2010; Lushchak, 2012; Denzoin Vulcano et al., 2013; García-Giménez et al., 2013; Lu, 2013; Giustarini et al., 2016; Scirè et al., 2019; Marí et al., 2020; Polonikov, 2020; Sestili and Fimognari, 2020; Silvagno et al., 2020; Bartolini et al., 2021). SARS-CoV-2 markedly decreases the levels of cellular thiols, essentially lowering the reduced form of GSH; and the use of antivirals that enhance activation of the Nrf2 transcription factor together with N-acetylcysteine administration restore GSH levels correcting the SARS-CoV-2-mediated impaired GSH metabolism (Aquilano et al., 2014; Khanfar and Al Qaroot, 2020; Sestili and Fimognari, 2020; Silvagno et al., 2020; Bartolini et al., 2021; Bourgonje et al., 2021; Fraternale et al., 2021; Kumar P. et al., 2022; Figure 4).

Alterations in the intracellular redox status are often associated with GSH depletion (Polonikov, 2020) and endogenous GSH deficiency, due either to decreased biosynthesis and/or increased consumption, is a marked contributor to the pathogenesis of numerous diseases *via* mechanisms including oxidative stress and inflammation. GSH deficiency is associated with age, a major risk factor for SARS-CoV-2 infection’s outcome (Grigoletto Fernandes et al., 2020; Polonikov, 2020; Sestili and Fimognari, 2020; Suhail et al., 2020; Yang X. et al., 2020; Yang J. et al., 2020; Schwalfenberg, 2021), sex, low intracellular GSH, cigarette smoking and presence of chronic diseases, among other factors, and GSH deficiency caused by any or all these factors may contribute to severe COVID-19 disease pathogenesis (Polonikov, 2020; Sestili and Fimognari, 2020; Silvagno et al., 2020; Suhail et al., 2020; Yang J. et al., 2020; Yang X. et al., 2020; Figure 1). Individuals 65 years and older with comorbidities, are more susceptible to become infected with SARS-CoV-2 and become critically ill and are more prone to develop ARDS and require mechanical ventilation, having a high 28-day mortality rate (Yang X. et al., 2020).

COVID-19 is clinically mild in most cases, severe cases develop pneumonia, and critical cases end with ARDS, sepsis, and multiple organ failure (Yang X. et al., 2020). COVID-19 sepsis is a serious problem in critically ill patients infected with SARS-CoV-2 (Beltrán-García et al., 2020; Coz Yataco and Simpson, 2020; De Candia et al., 2021). Sepsis is a systemic inflammatory response caused by excessive cytokine secretion, such as interleukin (IL)-6, IL-10, IL1β and TNF-α. Severe SARS-CoV-2 infection with high cytokine levels causes T-cell exhaustion characterized by high levels of programmed cell death protein 1 and low numbers of CD4^+^ and CD8^+^ cells (Diao et al., 2020; Lin et al., 2020; Poe and Corn, 2020). ROS may be important mediators of cellular injury during COVID-19 sepsis (Silvagno et al., 2020), either following macromolecular damage or by hindering extracellular and intracellular regulatory processes. Multiple organ failure including sepsis-induced cardiac dysfunction seem to be the result of numerous factors as overwhelming inflammation and nitric oxide synthesis impairment associated with mitochondrial dysfunction and increased oxidative stress (Cecchini and Cecchini, 2020). Excessive ROS production, associated with inflammation, induces oxidative stress. Oxidative stress is a major contributor to the high mortality rates associated with SARS-CoV-2 infections (Poljsak et al., 2013; Forcados et al., 2021; Lage et al., 2022). Immune cells use ROS to sustain their functions and need adequate levels of antioxidant defenses to avoid harmful effects caused by excessive ROS production, since accurate balance between ROS and intracellular antioxidants is essential for a normal function of the cell (Banjac et al., 2008; Liu et al., 2018).

L-cysteine is the rate-limiting substrate in the synthesis of intracellular GSH (Raftos et al., 2007; Whillier et al., 2009; Radtke et al., 2012). Although N-acetylcysteine (NAC) directly influences the pool of extracellular cystine and intracellular cysteine *via* a series of plasmatic redox reactions, in order to be effective, intracellular cysteine precursors must be designed to enter erythrocytes rapidly and use high activity enzymes within erythrocytes to liberate cysteine (Whillier et al., 2009; Radtke et al., 2012). NAC enhances extracellular cysteine and by using transport channels increases intracellular cysteine (Franco and Cidlowski, 2009; Aldini et al., 2018; Liu et al., 2018; Ulrich and Jakob, 2019; Pedre et al., 2021; Schwalfenberg, 2021). During oxidative stress, NAC will increase GSH synthesis (Franco and Cidlowski, 2009; Rushworth and Megson, 2014; Campolo et al., 2017). Without oxidative stress, cysteine and cystine appear to essentially mediate cellular stress *via* thiols other than GSH (Rahman and MacNee, 2000; Ashfaq et al., 2008; Sekhar et al., 2011a; Liu et al., 2018).

Individuals 60 years and older have lower plasma GSH levels and increased oxidative stress (Samiec et al., 1998). Individuals with diabetes have lower GSH levels compared to control subjects (Samiec et al., 1998; Sekhar et al., 2011b). Supplementation of cysteine and glycine in the diet can increase GSH levels and reduce oxidative stress in the elderly and persons with diabetes (Sekhar et al., 2011b; Tan et al., 2018). Elderly adults may also have reduced redox potential due to lower GSH levels (Samiec et al., 1998; Maher, 2005; Trachootham et al., 2008; Aquilano et al., 2014; McCarty and DiNicolantonio, 2015; Baş, 2018; Hajjar et al., 2018; Polonikov, 2020; Sestili and Fimognari, 2020). Lowered cellular redox status increases susceptibility to oxidative stress that may lead to cell death and virus release (Raftos et al., 2007; Hotchkiss et al., 2009; Circu and Aw, 2010; Kesarwani et al., 2013; He et al., 2017; Khomich et al., 2018; Chen et al., 2020; Sharifi-Rad et al., 2020; Zhang et al., 2020). The potential clinical use of antioxidants and antioxidant precursors in the treatment of COVID-19 needs to be seriously considered. GSH is paramount with respect to disease pathogenesis and individual response to COVID-19 infection; and enhancement of GSH levels can be a means for treating and preventing COVID-19 disease (Polonikov, 2020). As it was recently suggested, GSH depletion could be the Trojan horse of COVID-19 severity and mortality and elevating GSH levels in tissues may decrease COVID-19 severity and mortality rates (Khanfar and Al Qaroot, 2020).

## SARS-CoV-2 new therapeutic approaches

The prominence of the coronavirus disease 2019 (COVID-19) pandemic urges multidisciplinary strategies to control disease spread and prevent its complications (Labarrere and Kassab, 2021). SARS-CoV-2 and its massive cytokine storm primarily compromises the lungs causing acute respiratory distress syndrome also affecting the cardiovascular system aggravating atherosclerotic lesions leading to thromboembolic events and cell and tissue death (Ryu and Shin, 2021; Taoufik et al., 2021; Yuan et al., 2021). SARS-CoV-2 infects pulmonary type II alveolar cells because these cells express angiotensin-converting enzyme 2 (ACE2; Ryu and Shin, 2021; Taoufik et al., 2021; Yuan et al., 2021). SARS-CoV-2 ACE2-mediated host cell invasion is enhanced by the presence of heparan sulfate proteoglycans (HSPGs) consisting of a core protein bearing glycosaminoglycan carbohydrate chains (Souza-Fernandes et al., 2006; Davis and Parish, 2013; Kang et al., 2018; Clausen et al., 2020; De Pasquale et al., 2021). Virus protein ligands, like trimeric spike glycoprotein interact with cellular receptors, such as ACE2, and host proteases, like transmembrane protease serine 2 (TMPRSS2), participate in virus entry by proteolytically activating virus ligands (Sallenave and Guillot, 2020; Kalra and Kandimalla, 2021; Zhang Q. et al., 2021; Jackson et al., 2022). In the lungs, after entering in type II alveolar cells, SARS-CoV-2 infected cells become defective for surfactant production (Ghati et al., 2021) and release cytokines, like IL-8 among others, which in turn activate alveolar macrophages to release IL-1, IL-6, and TNF-α, that induce natural killer and dendritic cell differentiation and macrophage M1 polarization, enhancing the proinflammatory response with increased vasodilation causing neutrophil and activated T cell influx from the capillaries into the alveolus, all leading to the cytokine storm (Carcaterra and Caruso, 2021; Figure 2).

Alveolar macrophages, due to their polarization state toward M1 or M2 phenotypes, provoke different effects following SARS-CoV-2 infection. Hyperactivated M1 alveolar macrophages are taken over by SARS-CoV-2 allowing for viral infection and spread, while M2 alveolar macrophages can degrade the virus and limit its spread (Knoll et al., 2021; Lv et al., 2021). Neutrophils produce ROS and proteinases, causing further destruction of healthy type II cells; as a result, surfactant production decreases markedly, which in turn causes alveolar fluid accumulation leading to alveolar collapse and ARDS (Matthay and Zemans, 2011; Carcaterra and Caruso, 2021). Due to the exhaustion of cellular and extracellular GSH caused by numerous GSH-consuming pathways the severe inflammation and oxidative stress triggered by the viral infection steals GSH from crucial functions like NO-dependent vasodilatation, disallowing the patient of being protected from an inflammation that can become fatal. Based on the previous discussion, administration of antioxidants or Nrf2 inducers are potential viable therapies for viral-induced diseases, like respiratory infections and infections associated with reduced cellular antioxidant capacity (Komaravelli and Casola, 2014). A high neutrophil to lymphocyte ratio found in critically ill patients with COVID-19 is associated with excessive ROS levels, that promote a cascade of biological events driving pathological host responses. Since ROS induce tissue damage, thrombosis and red blood cell dysfunction that contribute to COVID-19 disease severity, administration of free radical scavengers could be beneficial for the most vulnerable patients (Laforge et al., 2020).

Toll-like receptors (TLRs) play a key role in microorganism and viral particle recognition and activation of the innate immune system (Sasai and Yamamoto, 2013; Kawasaki and Kawai, 2014; McClure and Massari, 2014; Sartorius et al., 2021; Manik and Singh, 2022). TLR pathway activation leads to secretion of pro-inflammatory cytokines, like interleukin (IL)-1, IL-6, and tumor necrosis factor-α, as well as type 1 interferon. TLRs can be localized either on the cell surface (TLR-1, -2, -4, -5, -6, -10) or in the endosome compartment (TLR-3, -7, -8, -9; Sasai and Yamamoto, 2013; Kawasaki and Kawai, 2014; Sartorius et al., 2021). TLRs-2, -3, -4, -6, -7, -8, and -9 are potentially important in COVID-19 infection (Onofrio et al., 2020; Khanmohammadi and Rezaei, 2021; Sariol and Perlman, 2021). TLR1/2/6 activation and subsequent signal transduction may be in part responsible for clinical immunopathological manifestations found in patients infected with COVID-19 (Gadanec et al., 2021). Interactions between TLR1/6 and the S-protein may participate in immunopathology as a result of unregulated TLR activation (Kawasaki and Kawai, 2014; Gadanec et al., 2021). SARS-CoV-2 may activate TLR4 in the heart and lungs causing aberrant TLR4 signaling favoring the proinflammatory MyD88-dependent (canonical) pathway instead of the alternative TRIF/TRAM-dependent anti-inflammatory and interferon pathway (Aboudounya and Heads, 2021). TLR4-mediated recognition of S protein may initiate receptor dependent internalization and explain SARS-CoV-2 infection in patients and cells lacking or deficient in ACE2 expression (Aboudounya and Heads, 2021; Gadanec et al., 2021). Viral proteins as well as host damage-associated molecular patterns, that accumulate following cellular stress during viral infection, were linked to TLR4 activation, with uncontrolled TLR4 activation being associated with severe disease (Olejnik et al., 2018). TLR4 activation in platelets whether by pathogen- (viremia) or damage-associated molecular patterns induces a prothrombotic and proinflammatory state (Schattner, 2019). SARS-CoV-2 spike glycoprotein binds TLR4 and activates TLR4 signaling increasing cell surface expression of ACE2 facilitating entry (Aboudounya and Heads, 2021). Activation of endosomal TLR7/8 during SARS-CoV-2 may increase the inflammatory response resulting in severe and potentially lethal immunopathological effects in COVID-19 patients as consequence of the simultaneous release of pro-inflammatory cytokines and chemokines. TLR signaling molecules, like mitogen-activated protein kinases (MAPK) and phosphoinositide 3-kinase (PI3K)/protein kinase B (Akt), play fundamental roles in TLR-mediated cell proliferation and survival *via* reducing apoptosis and increasing time for viral replication (Li et al., 2010; Aboudounya and Heads, 2021) and could facilitate increased viral replication. The expression of CD14, TLR2 and 4 in human alveolar type I and II cells (Thorley et al., 2011) suggests that they most probably participate in SARS-CoV-2 infection and COVID-19 disease. Ten human TLRs that signal *via* 4 adaptor proteins and 2 initial kinases activate distal kinases that subsequently regulate transcription factors such as NFκB and activator protein 1 (AP-1), that control gene expression. Posttranslational modifications of ROS-mediated kinase activity most probably contribute to the diversity and intensity of gene expression following microbial activation of innate immunity (Kolls, 2006).

SARS-CoV-2 mainly destroys pulmonary surfactant-secreting type II alveolar cells (Wang et al., 2021a), that normally decrease the air/tissue surface tension and block TLR4 in the lungs, promoting ARDS and inflammation. TLR4 activation, aberrant TLR4 signaling, and hyperinflammation may explain SARS-CoV-2-induced myocarditis and multiple-organ injury in COVID-19 patients (Aboudounya and Heads, 2021). Augmented activation of TLR4 increases oxidative stress and the generated ROS participate in signaling events downstream of TLRs. TLR4 activation may lead to ROS signaling *via* direct interaction between TLR4 and NADPH oxidase (Gill et al., 2010; Pushpakumar et al., 2017). TLR1, TLR2 and TLR4 activation results in augmented mitochondrial ROS production following recruitment of mitochondria to macrophage phagosomes, leading the way to increased mitochondrial and cellular ROS generation (West et al., 2011). ROS can oxidize cysteine residues allowing formation of disulfide bridges with one another or with GSH leading to S-glutathionylation. ROS can be inactivated by antioxidants such as GSH. The link between oxidation and inflammation is complex, going from fine-tuned signaling by ROS during TLR4 activation that leads to active mobilization of damaged-associated molecular patterns, to cellular injury from redox stress that leads to damaged-associated molecular patterns release triggering TLR4-mediated inflammation and organ injury (Gill et al., 2010; Pushpakumar et al., 2017). Neutralization of oxidation radicals becomes paramount in SARS-CoV-2-mediated cellular and tissue damage.

**Figure 5:**
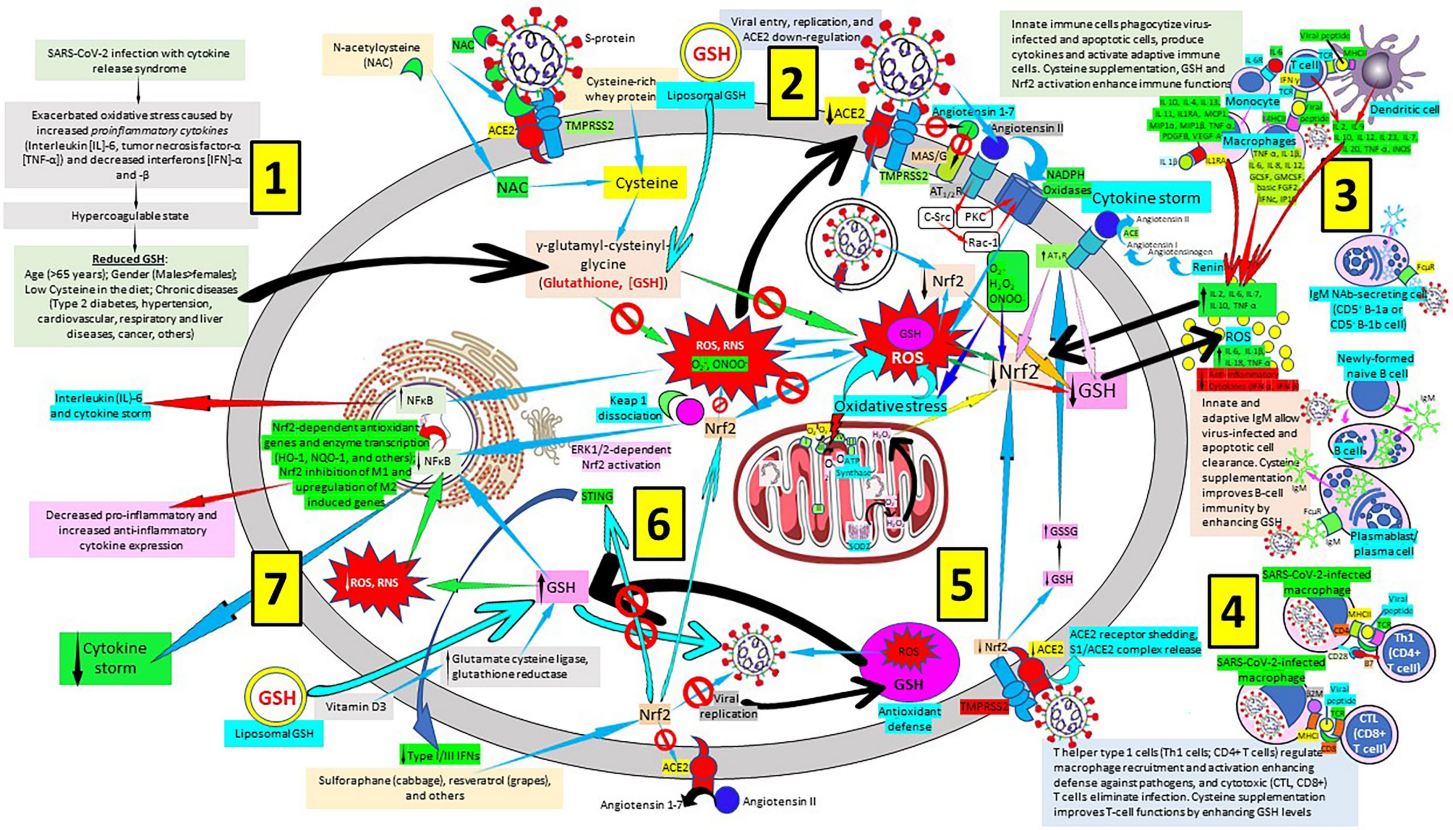
Severe acute respiratory syndrome coronavirus 2 (SARS-CoV-2)-related glutathione (GSH) cellular depletion, repletion treatment options and a multiweapon defense approach. [1] SARS-CoV-2 exacerbates oxidative stress, inflammation and coagulation, reducing GSH levels mainly in hosts with added risk factors. [2] SARS-CoV-2 invades host cells through virus protein ligands, like trimeric spike glycoprotein interacting with cellular receptors like angiotensin converting enzyme 2 (ACE2), and host proteases, like transmembrane protease serine 2 (TMPRSS2), that proteolytically activates virus ligands, promoting virus entry, replication and ACE2 down-regulation; N-acetylcysteine (NAC) can block this interaction. SARS-CoV-2-related ACE2 downregulation facilitates angiotensin II/angiotensin types 1 and 2 receptor (AT1/2R)-mediated nicotinamide adenine dinucleotide phosphate (NADPH) oxidase activation and reactive oxygen species (ROS) production; and inhibits interaction of angiotensin 1–7 and MAS1 oncogene/G protein-coupled (MAS/G) receptor. [3] A multiweapon approach includes enhancing host response to viral particles and peptides by monocytes/macrophages and T-cells, as well as innate and adaptive B-cell/plasma cells producing antibodies to reduce cytokine production and the subsequent cytokine storm. Reactive oxygen species (ROS) cell production enhances proinflammatory cytokine release while reducing anti-inflammatory cytokines. [4] Modulation of CD4+ and CD8+ T cells will facilitate SARS-CoV-2 removal. [5] SARS-CoV-2 binding and cleavage of ACE2 receptor leads to shedding of host ACE2 receptor contributing to the loss of ACE2 function and systemic release of S1/ACE2 complex. SARS-CoV-2 reduces Nrf2 and GSH allowing ROS and RNS to damage the cell. [6] Liposomal GSH (see [2], vitamin D3 by increasing glutamate cysteine ligase and glutathione reductase activity), and N acetyl cysteine (NAC), see [2] participate in GSH synthesis. Increased intracellular GSH reduces ROS and reactive nitrogen species (RNS), as well as NF-κB activation. Sulforaphane and resveratrol enhance Nrf2 production and Nrf2 negatively regulates the endoplasmic-reticulum-resident protein stimulator of interferon genes (STING) reducing interferon secretion. Increased antioxidant defense (cystine, cysteine, NAC, liposomal GSH, vitamin D3, sulforaphane, resveratrol, and others) reestablishes cell homeostasis [7]. Increased nuclear factor-κB (NF-κB) activity enhances interleukin (IL)-6 secretion and cytokine storm, while decreased nuclear NF-κB allows activation of nuclear factor erythroid 2-related factor2 (Nrf2)-dependent antioxidant genes and enzyme transcription (HO-1, NQO-1, and others); Nrf2 inhibition of M1 and upregulation of M2 induced genes; decreased pro-inflammatory and increased anti-inflammatory cytokine expression; and decreased cytokine storm. C-Src, proto-oncogene tyrosine-protein kinase sarcoma; PKC, protein kinase C; Rac-1, Ras-related C3 botulinum toxin substrate 1; IL1RA, IL1 receptor antagonist; MCP1, monocyte chemoattractant protein-1; MIP, macrophage inflammatory protein; PDGFB, Platelet Derived Growth Factor B; VEGF-A, vascular endothelial growth factor A; iNOS, inducible nitric oxide synthase; TCR, T-cell receptor; MHCI/II, major histocompatibility complex class I/II; GCSF, granulocyte colony-stimulating factor; GMCSF, granulocyte-macrophage colony-stimulating factor; FGF, fibroblast growth factor; IP10, interferon gamma-induced protein 10; NAb, natural antibody; SOD, superoxide dismutase; HO-1, heme oxygenase-1; NQO1, NAD(P) H quinone dehydrogenase 1; β2M, β2 microglobulin; GSSG, glutathione disulfide; Keap 1, Kelch-like ECH-associated protein 1; ERK, extracellular signal-regulated protein kinase; FcμR, Fcμ receptor; IgM, immunoglobulin M.

As we previously published, a multiweapon approach is needed to successfully combat SARS-CoV-2 and COVID-19 disease (Labarrere and Kassab, 2021), involving vaccines (Jin et al., 2022) especially vaccines that selectively and efficiently induce antibodies that target the SARS-CoV-2 receptor binding domain (Robbiani et al., 2020), pattern recognition proteins such as surfactant proteins A and D (Arroyo et al., 2021; Ghati et al., 2021; Wang et al., 2021a; DePietro and Salzberg, 2022; Labarrere and Kassab, 2022), modulators of mannose binding lectin, C1q, C-reactive protein (Tang et al., 2011; Torzewski et al., 2020; Labarrere and Kassab, 2021; Ringel et al., 2021), and IgM natural antibodies, TLR inhibitors, modulators of cellular components (neutrophils, basophils, eosinophils, mast cells, monocytes, macrophages, dendritic cells, regulatory T cells, natural killer cells) of innate immunity, cellular components of both innate and adaptive immune systems (γδ T cells, natural killer T cells), soluble constituents of adaptive immunity (polyreactive IgM antibodies to the viral disease, among others), and cellular components of adaptive immunity (T cell subsets like Th1 CD4+ T cells, cytotoxic CD8+ T cells, Th2 cells, Th17 cells, Th9 cells), viral replication inhibitors, renin-angiotensin system inhibitors (Williams, 2021) and human recombinant soluble ACE2 (Abd El-Aziz et al., 2020), as well as heparin and glycosaminoglycan antithrombotics (Magnani, 2021), among others. Sadly, there are no effective antivirals and vaccines to definitively treat or prevent COVID-19. Globally launched clinical trials like the European study DISCOVERY showed that antiviral drugs (remdesivir, lopinavir and ritonavir in combination, ritonavir given with or without interferon beta and hydroxychloroquine) are unable to efficiently attack COVID-19 progression (Ader et al., 2022). Although a recent trial has shown to be beneficial when antiviral treatment is introduced early during the disease before hospitalization than later in the course of the disease, there is an urgent need for early therapies to reduce the risk of disease progression, prevent transmission, and be widely distributed to meet the worldwide demand (Gottlieb et al., 2022; Heil and Kottilil, 2022). Here we emphasize the role of Nrf2 activators and the vital role of antioxidants like the GSH system in prevention against oxidative stress and cell and tissue damage (Cuadrado et al., 2020) in SARS-CoV-2 infection and COVID-19 disease (Figure 5).

Since oxidative stress plays an important role in the pathogenesis of viral-associated cardiovascular and lung diseases, antioxidant intervention would be a rational approach to use for treating lower respiratory tract infections (Komaravelli and Casola, 2014) and balancing oxidative damage by enhancing antioxidant defense (Banjac et al., 2008; Chen et al., 2020; Sharifi-Rad et al., 2020; Perła-Kaján and Jakubowski, 2022) could be a major strategy for a successful intervention against SARS-CoV-2 infection and COVID-19 disease. Since SARS-CoV-2 activates mitochondrial ROS-mediated feedback loops that produce long-term changes in the redox status and endothelial function of the host, leading to cardiovascular disease and lung injury (Chang et al., 2021), and endothelial cells are key players in inflammatory pathologies, such as acute respiratory distress syndrome, thrombosis, and atherosclerosis; the use of pro-GSH molecules (Fraternale et al., 2006) and/or glutathione precursors like N-acetyl cysteine (NAC), glutamine, cysteine (cystine) and glycine, as well as nuclear factor erythroid 2 p45–related factor 2 (Nrf2) inducers like sulforaphane can enhance glutathione production and increase nuclear Nrf2 translocation and antioxidant response element (ARE) transcription (Komaravelli and Casola, 2014; Atefi et al., 2020; Poe and Corn, 2020; Dominari et al., 2021; Obayan, 2021; Di Marco et al., 2022). Nrf2, a member of the “cap’n’collar” family of basic region–leucine zipper transcription factors involved in transcription of antioxidant genes in response to xenobiotic stress, is also a critical regulator of cellular oxidative stress in sepsis (Kolls, 2006) and a regulator of exacerbated proinflammatory cytokine release (cytokine storm) and loss of T lymphocytes (leukopenia) that characterize the most aggressive presentation of SARS-CoV-2-mediated COVID-19 infection (Kobayashi et al., 2016; Cuadrado et al., 2019; Olagnier et al., 2020; Calabrese et al., 2021; Herengt et al., 2021). By regulating glutathione S-transferase (GST) and intracellular glutathione (GSH) levels, Nrf2 controls the level of ROS in the cell (Kolls, 2006; Lushchak, 2012; Aquilano et al., 2014; Tu et al., 2019; Bartolini et al., 2021); and a multifaceted anti-inflammatory strategy based on pharmacological activation of Nrf2 and enhancement of GSH precursors like bonded cysteine or NAC can be deployed against the virus (Cuadrado et al., 2019; Olagnier et al., 2020; Emanuele et al., 2021; Fratta Pasini et al., 2021; Wong et al., 2021). Since Nrf2 participates in the resolution of inflammation by repressing genes for proinflammatory cytokines IL-6 and IL-1β (Kobayashi et al., 2016; Cuadrado et al., 2020) and pharmacological activation of Nrf2 might also limit NF-κB-mediated pulmonary inflammation caused by SARS-CoV-2 infection (Cuadrado et al., 2019, 2020; Chang et al., 2021; Emanuele et al., 2021; Fratta Pasini et al., 2021). NRF2 inducers, like sulforaphane modify cysteine sensors of Keap1 and inactivate its repressor function. The liberation of Nrf2 from Keap1 allows Nrf2 accumulation and translocation to the nucleus (Cuadrado et al., 2019; McCord et al., 2020; Perła-Kaján and Jakubowski, 2022). In the nucleus, following heterodimer complex formation with transcription factors, like small Maf proteins (G/F/K) and c-Jun, Nrf2 complexes bind to the antioxidant response element (ARE), a regulatory enhancer region within gene promoters that upregulate antioxidant and anti-inflammatory defense processes (Cuadrado et al., 2019; McCord et al., 2020; Perła-Kaján and Jakubowski, 2022).

Since SARS-CoV-2 mediates Nrf2 suppression and limits host anti-inflammatory response (Cuadrado et al., 2020; McCord et al., 2020; Olagnier et al., 2020; Emanuele et al., 2021; Fratta Pasini et al., 2021), targeting Nrf2 is therefore essential for the treatment of diseases characterized by enhanced oxidative stress and inflammation, such as aging and COVID-19-induced pneumonia and ARDS (Lewis et al., 2010; Lee, 2018; Robledinos-Antón et al., 2019; Schmidlin et al., 2019; Lin and Yao, 2020). Nrf2 activation suppresses ROS in antigen-presenting dendritic cells enhancing their capacity to interact with and promote the transformation of naïve CD8 T cells into cytotoxic T lymphocytes enabling cytotoxic T-cells to eliminate virally infected cells (Kesarwani et al., 2013; Moro-García et al., 2018; Calabrese et al., 2021; Emanuele et al., 2021). Nrf2 activation regulates antioxidant responses to modify cellular redox states from predominantly pro-oxidant to antioxidant, and, in an antioxidant environment, macrophage phenotypes shift from M1 pro-inflammatory to M2 anti-inflammatory, reducing the probability of cytokine storms, ARDS, and lethality (Tan et al., 2016; Bousquet et al., 2020; Calabrese et al., 2021). Cytoprotective effects against viruses like SARS-CoV-2 could be enhanced by sulforaphane, an isothiocyanate abundant in cruciferous vegetables, since sulforaphane has been found to be a powerful activator of the Nrf2 pathway by increasing Nrf2-regulated cellular antioxidant response such as induction of NAD(P)H: quinone oxidoreductase 1, glutamate-cysteine ligase (γ-glutamyl cysteine synthetase) and glutathione (Theodore et al., 2008; Lewis et al., 2010; Schmidlin et al., 2019; Bousquet et al., 2020; Cuadrado et al., 2020; Mahn and Castillo, 2021). Nrf2 activators like sulforaphane have a potential role with dual antiviral and anti-inflammatory properties in the management of viral pneumonia, a serious complication in COVID-19 disease (Bousquet et al., 2020; Cuadrado et al., 2020; Lin and Yao, 2020; Emanuele et al., 2021; Fratta Pasini et al., 2021). Nrf2-interacting nutrients can equilibrate insulin resistance and have a significant effect upon COVID-19 severity. It is then possible that intake of these nutrients may re-establish an optimal natural balance for the Nrf2 pathway and mitigate COVID-19 severity (Bousquet et al., 2020). The enhancement of Nrf2 transcription with sulforaphane or melatonin could benefit patients with “LONG COVID” (Post-Acute Sequelae of SARS-CoV-2 or Post-COVID-Syndrome), a significant proportion (approximately 40%) of individuals with COVID-19 experiencing a variety of symptoms (loss of smell and/or taste, fatigue, cough, aching pain, “brain fog,” insomnia, shortness of breath, and tachycardia) after 12 weeks (Jarrott et al., 2022; Ordonez et al., 2022). Multi-omics studies revealed that SARS-CoV-2 infection provokes significant changes in numerous metabolites including those impacting on virus propagation (one-carbon metabolism) and GSH synthesis (amino acids glutamic acid, cysteine and glycine) that could be used as early prognosis biomarkers in COVID-19 at diagnose to predict severe COVID-19 and “LONG COVID” (Doğan et al., 2021; Li C.-X. et al., 2022; Perła-Kaján and Jakubowski, 2022; Valdés et al., 2022). Therapeutic interventions aimed at normalizing GSH and Nrf2 might provide a promising approach to combat the COVID-19 pandemic.

Augmented oxidative stress secondary to increased levels of interleukin-6 and tumor necrosis factor-α in addition to decreased levels of interferons α and β are primarily believed to be the drivers of the disease process (Guloyan et al., 2020). Since it was shown that glutathione (GSH) inhibits viral replication and decreases IL-6 levels, it was suggested that liposomal GSH could be beneficial in COVID-19 patients characterized by SARS-CoV-2-induced cytokine storm and redox imbalance (Guloyan et al., 2020). SARS-CoV-2 binds to the ACE2 receptor and induces down regulation of NRF2, which leads to inhibition of GSH release. This leads to elevated inflammatory cytokines, elevated ROS, and recruitment of immune cells. The importance of thiol-reactive molecules like NAC and GSH in SARS-CoV-2 infectivity has been shown recently (Murae et al., 2022). NAC and GSH directly suppress spike protein receptor-binding domain-ACE2 binding functions of various SARS-CoV-2 variants. An intramolecular disulfide bridge in the receptor-binding domain of the SARS-CoV-2 spike protein between Cys-488 and Cys-480, considered to be important for ACE2-binding, results directly inhibited by NAC and GSH and these compounds could be used effectively against SARS-CoV-2 cell viral entry and infection (Murae et al., 2022). GSH was shown to be the main inhibitor in the active site of the main protease (M^pro^), the essential protein for virus invasion, and cysteine (Cys300) glutathionylation inhibits M^pro^ activity by blocking its dimerization supporting the use of GSH in COVID-19 patients (Davis et al., 2021; Linani et al., 2022). GSH deficiency has been associated with increased ROS and more severe clinical COVID-19 (Guillin et al., 2019; Derouiche, 2020; Grigoletto Fernandes et al., 2020; Guloyan et al., 2020; Polonikov, 2020; Sestili and Fimognari, 2020; Silvagno et al., 2020; Suhail et al., 2020; Bourgonje et al., 2021; Forcados et al., 2021; Kozlov et al., 2021; Pérez de la Lastra et al., 2021; Kumar P. et al., 2022). SARS-CoV-2 affects intracellular GSH levels by decreasing intracellular NRF2 function, that plays a key role in protecting cells from oxidative damage by upregulating GSH production (Rahman and MacNee, 2000; Guloyan et al., 2020; Polonikov, 2020; Sharifi-Rad et al., 2020; Silvagno et al., 2020; Bartolini et al., 2021). In stressed cells NRF2 is released and taken from the cytoplasm into the nucleus by karyopherins (Theodore et al., 2008; Sims et al., 2013; Bousquet et al., 2020; Derouiche, 2020; Guloyan et al., 2020). Coronavirus inhibits karyopherin-mediated nuclear import decreasing GSH production (Sims et al., 2013; Guloyan et al., 2020). In the setting of SARS-CoV-2, COVID-19 and oxidative stress, patients with comorbidities may have altered levels of glutamate-cysteine ligase and GSH synthetase, the enzymes participating in GSH synthesis. Therefore, it is reasonable using supplementation of liposomal glutathione, instead of the N-acetylcysteine or bonded cysteine utilized as precursors for GSH cell synthesis, since patients with deficient levels of glutamate-cysteine ligase and GSH synthetase will not be able to use N-acetylcysteine or bonded cysteine as substrates to synthesize their own GSH. Replenishing the nutritional status of the host by increasing vital amino acids such as cysteine to enhance GSH levels and selenium to improve selenium deficiency and facilitate selenoprotein (GSH peroxidases, thioredoxin reductases) expression can inhibit oxidative stress, modulating inflammation, suppressing endothelial dysfunction, and protecting vascular cells against apoptosis and calcification (He et al., 2017; Guillin et al., 2019; Seale et al., 2020; Taylor and Radding, 2020; Martinez et al., 2022). The demonstration that a combination of glycine and N-acetylcysteine supplementation rapidly improves GSH deficiency, oxidative stress and oxidant damage has implications for considering the GSH importance in combating COVID-19 infected patients warranting further investigations (Kumar P. et al., 2022). Enzymes involved in GSH biosynthesis and function like γ-glutamyl-cysteine ligase and glutathione synthetase are completely dependent on ATP and require magnesium as a cofactor (Bani Younes et al., 2020; Tang et al., 2020; Arancibia-Hernández et al., 2022). Additionally, γ-glutamyl-transpeptidase uses magnesium as an enzyme activator (Arancibia-Hernández et al., 2022). Magnesium supplementation improves mitochondrial function and increases the content of GSH in those organelles (Liu et al., 2019; Mohammadi et al., 2020). Furthermore, magnesium sulfate was effective as a treatment for preeclampsia, significantly promoting GSH production and suppressing ROS generation (Kawasaki et al., 2019; Arancibia-Hernández et al., 2022). Recent studies have suggested that serum magnesium levels of critically ill patients deserve attention (Bani Younes et al., 2020; Iotti et al., 2020) and could not only prevent SARS CoV-2 infection, reduce severity of COVID-19 symptoms and facilitate disease recovery (Trapani et al., 2022) but benefit enzymatic activity of the GSH pathway in COVID-19. Molecules of nutritional value with antioxidant properties besides GSH, like selenium, zinc and polyphenols, are important in the immune response against SARS-CoV-2 that occurs primary in the lungs (Pérez de la Lastra et al., 2021) in critically ill patients with severe COVID-19 acute respiratory distress syndrome (Notz et al., 2021). The value of selenium upon glutathione peroxidase 1 activity and oxidative stress mitigation in SARS-CoV-2 infection and COVID-19 disease has been clearly emphasized recently (Seale et al., 2020; Fakhrolmobasheri et al., 2021) and can prevent atherosclerosis progression in SARS-CoV-2 infection. The recent demonstration of elevated superoxide dismutase, GSH peroxidase, and total antioxidant capacity in COVID-19 outpatients compared to controls could be interpreted as a response to excessive COVID-19-related oxidative stress (Golabi et al., 2022). Adequate levels and function of GSH and selenoproteins can prevent worsening of acute respiratory distress syndrome and atherosclerosis, two main causes of morbimortality in SARS-CoV-2 infection and COVID-19 disease.

## Conclusion

COVID-19 is a historic challenge to the fields of research, infectious disease, and global healthcare (Hunter et al., 2022). The demand for detailed analysis of COVID-19 pathogenesis and clinical course is paramount. The unprecedented awareness of a rapidly spreading pandemic disease such as COVID-19 brings an opportunity to enhance international collaboration in the scientific community. As new variants like the omicron (Abdool Karim and Abdool, 2021; Callaway and Ledford, 2021) and others (Markov et al., 2022) appear, besides new vaccine trials continuously ongoing, physicians have been encouraged to utilize various treatments with established efficacy in similar viral or bacterial illnesses that also cause bilateral pneumonia and ARDS, as SARS-CoV-2 does. Here we present the antioxidant GSH as a potential unexplored way for further investigation as intervention for COVID-19, since GSH levels are correlated with disease severity and lung damage supporting the participation of GSH in disease outcome (Kryukov et al., 2021; Singh et al., 2022). Enhancing GSH, mainly through NAC, GSH precursors or pro-GSH compound administration, becomes a potential treatment option for SARS-CoV-2 infection and COVID-19 disease by reducing oxidative stress and cytokine expression especially in diabetic patients at risk of more severe disease (Singh et al., 2022). Whey protein concentrate ameliorates lung damage and inhibits lung furin activity targeting SARS-CoV-2 S1/S2 site cleavage and SARS CoV-2 spike protein-angiotensin converting enzyme binding and could be used to protect against COVID-19 inhibiting SARS-CoV-2 cell entry (Tufan et al., 2022). A combination of vitamin D and L-cysteine administration significantly augmented GSH levels and lowered oxidative stress and inflammation (Jain et al., 2018; Jain and Parsanathan, 2020). Maintaining an adequate GSH redox status and 25-hydroxy-vitamin D levels will have the potential to reduce oxidative stress, enhance immunity and diminish the adverse clinical consequences of COVID-19 especially in African American communities having glucose-6-phosphate dehydrogenase deficiency, enzyme necessary to prevent GSH exhaustion and depletion (Jain and Parsanathan, 2020; Jain et al., 2020). We propose that enhancement of the reduced form of GSH will reduce the body’s oxidation and inflammation associated with SARS-CoV-2 infection and COVID-19 disease (Karkhanei et al., 2021). Maintaining GSH levels using therapies that do not deplete the body’s GSH (Sestili and Fimognari, 2020) would be the best choice. In a patient that is overloaded with cytokine storm, the best way to fortify the immune system would be to supply it with reduced GSH, since reduced GSH is already able to provide reducing equivalents from its thiol group. This is particularly relevant when we consider GSH pathways, as well as their transcriptional regulator Nrf2, for proliferation, survival and function of T cells, B cells and macrophages (Muri and Kopf, 2021). The value of GSH and nutritional strategies like amino acids, vitamins, minerals, phytochemicals, sulforaphane to enhance cellular Nrf2, and other supplements used to restore GSH levels (Minich and Brown, 2019; Hermel et al., 2021) as adjunct treatments for SARS-CoV-2 infection needs to be further emphasized. Reestablishing the cellular metabolic homeostasis in SARS-CoV-2 infection and COVID-19 disease especially in the lungs, could become paramount to balance altered innate and adaptive immunity and cell function and reduce morbimortality (Hsu et al., 2022; Li S. et al., 2022). COVID-19 of the respiratory system appears to be a complex disease that may resist finding a single silver bullet intervention (Brosnahan et al., 2020). A multi-weaponry approach (Table 1) that includes global vaccine availability distributed without the greedy and selfish attitude of pharmaceutical company executives and shareholders or politicians, needs to bear in mind that “no one is safe until everyone is safe” (Hunter et al., 2022).

**Table 1:** Multi-weaponry approach involving glutathione (GSH) enhancers, nuclear factor erythroid 2 p45–related factor 2 (Nrf2) activators, toll-like receptor (TLR) inhibitors/immunomodulators, C-reactive protein (CRP) level reduction, natural and immune immunoglobulin M (IgM) enhancement and immune cell function recovery against SARS-CoV-2 infection and COVID-19 disease.

	Proinflammatory effects	Anti-inflammatory effects	Treatment effects on SARS-CoV-2/COVID-19
GSH Enhancers (N-acetylcysteine [NAC], glutamine, cysteine [cystine], glycine)	GSH is fundamental to sustain an adequate function of the immune system, particularly affecting the lymphocyte activity since low GSH levels inhibit T-cell proliferation and immune response (Dröge and Breitkreutz, 2000; Ghezzi, 2011; Moro-García et al., 2018; Kelly and Pearce, 2020; Khanfar and Al Qaroot, 2020; Shyer et al., 2020). GSH levels in macrophages, directly affect the Th1/Th2 cytokine response (Fraternale et al., 2006). GSH is capable of scavenging ROS through Nrf2-mediated heme oxygenase-1 induction and enhancing M1-like macrophage polarization regulation, showing that GSH may be a useful strategy to increase the human defense system (Mittal et al., 2014; Kwon et al., 2019; Funes et al., 2020). GSH increases activation of cytotoxic T cells *in vivo*, and adequate functioning of T lymphocytes and other cells depends upon cellular supplies of cysteine (Edinger and Thompson, 2002; Garg et al., 2011; Levring et al., 2015)	GSH inhibits production of most inflammatory cytokines, and it is needed to keep an adequate interferon gamma production by dendritic cells, essential for intracellular pathogen host defense (Ghezzi, 2011; Lee and Ashkar, 2018; Calder, 2020; Fraternale et al., 2021). The principal function of endogenous GSH is not to limit inflammation but to fine-tune the innate immune response to infection (Diotallevi et al., 2017; De Flora et al., 2020; Silvagno et al., 2020; Ferreira et al., 2021)	Administration of free radical scavengers could benefit the most vulnerable SARS-CoV-2-infected patients (Laforge et al., 2020). Many antioxidants like *GSH, and NAC* inhibit viral replication (Fraternale et al., 2006). GSH precursors like NAC, glutamine, cysteine (cystine) and glycine, and Nrf2 inducers like sulforaphane can enhance GSH production and increase nuclear Nrf2 translocation and antioxidant response element (ARE) transcription (Komaravelli and Casola, 2014; Atefi et al., 2020; Poe and Corn, 2020; Dominari et al., 2021; Obayan, 2021; Di Marco et al., 2022). Since GSH inhibits viral replication and decreases IL-6 levels, liposomal GSH could benefit COVID-19 patients having SARS-CoV-2-induced cytokine storm and redox imbalance (Guloyan et al., 2020). NAC and GSH directly suppress spike protein receptor-binding domain-ACE2 binding functions of various SARS-CoV-2 variants (Murae et al., 2022)
Nrf2 Activators (Sulforaphane, melatonin)	Nrf2 activation suppresses ROS in antigen-presenting dendritic cells enhancing their capacity to interact with and promote the transformation of naïve CD8 T cells into cytotoxic T lymphocytes enabling cytotoxic T-cells to eliminate virally infected cells (Kesarwani et al., 2013; Moro-García et al., 2018; Calabrese et al., 2021; Emanuele et al., 2021)	Nrf2 activation regulates antioxidant responses to modify cellular redox states from predominantly pro-oxidant to antioxidant, and, in an antioxidant environment, macrophage phenotypes shift from M1 pro-inflammatory to M2 anti-inflammatory, reducing the probability of cytokine storms, ARDS, and lethality (Tan et al., 2016; Bousquet et al., 2020; Calabrese et al., 2021)	Antioxidants (GSH, GSH enhancers) or Nrf2 inducers (sulforaphane, melatonin) are potential viable therapies for viral-induced diseases; Nrf2 activators like sulforaphane have a potential role with dual antiviral and anti-inflammatory properties in the management of COVID-19 pneumonia (Bousquet et al., 2020; Cuadrado et al., 2020; Lin and Yao, 2020; Emanuele et al., 2021; Fratta Pasini et al., 2021) and LONG COVID (Jarrott et al., 2022; Ordonez et al., 2022)
Toll-like receptors (TLRs)	TLR4 in the heart and lungs causing aberrant TLR4 signaling favors the proinflammatory MyD88-dependent (canonical) pathway instead of the alternative TRIF/TRAM-dependent anti-inflammatory and interferon pathway (Aboudounya and Heads, 2021). TLR4 activation in platelets whether by pathogen- (viremia) or damage-associated molecular patterns induces a prothrombotic and proinflammatory state (Schattner, 2019). Activation of endosomal TLR7/8 during SARS-CoV-2 may increase the inflammatory response resulting in severe and potentially lethal immunopathological effects in COVID-19 patients as consequence of the simultaneous release of pro-inflammatory cytokines and chemokines (Dai et al., 2022)	TLRs play a key role in microorganism and viral particle recognition and activation of the innate immune system and although pathogen-associated molecular pattern (PAMP) recognition by TLRs is crucial for host defense responses to pathogen infection, *aberrant activation of TLR signaling* by PAMPs, *mutations of TLR signaling molecules*, and *damage-associated molecular patterns (DAMPs)-mediated TLRs signaling activation* are responsible for the development of chronic inflammatory diseases (Sasai and Yamamoto, 2013; Kawasaki and Kawai, 2014; McClure and Massari, 2014; Sartorius et al., 2021; Manik and Singh, 2022)	GSH and GSH enhancers could neutralize oxidation radicals generated during TLR-mediated mitochondrial ROS production and directly affect SARS-CoV-2-mediated cellular and tissue damage (Aboudounya and Heads, 2021); TLR inhibitors/immunomodulators could become promising treatments for severe COVID-19 (Jung and Lee, 2021; Dai et al., 2022)
C-reactive protein	*Pentameric native (n)CRP*-FcɣRI/FcɣRIIa: increases inflammatory cytokine release; nCRP-FcɣRIIb maintains a predominant anti-inflammatory effect; *non-native (nn)CRP* enhances inflammation and complement activation; induces atherogenesis; mostly proinflammatory; *monomeric (m)CRP* promotes chemotaxis; increases IL-8, MCP-1 and nitric oxide; induces ROS; mCRP-FcɣRIII induce inflammation; promotes adhesion molecule expression, thrombosis and atherogenesis (Labarrere and Kassab, 2021)	*Pentameric nCRP* bound to phosphorylcholine (PC) or lysoPC-apoptotic cells, C1q and factor H: enhance phagocytosis; nCRP-FcɣRs: M2 response; *nnCRP* binds atherogenic LDL, reduces foam cell formation and could also be atheroprotective; *mCRP* is mainly proinflammatory and not anti-inflammatory (Labarrere and Kassab, 2021)	Binding of CRP to SARS-CoV-2 virus and/or the cell membrane can impair subsequent virus attachment and entry into the cell (Labarrere and Kassab, 2021). CRP apheresis could reduce CRP levels and inflammation (Ringel et al., 2021; Torzewski et al., 2020), and reduced GSH, which has the anti-inflammation and anti-oxidation effects, can significantly decrease the plasma concentrations of CRP (Tang et al., 2011)
Natural (innate) and immune (adaptive) immunoglobulin M (IgM)	*Lack of innate and adaptive IgM* allows cell necrosis and inflammation and prevents apoptotic cell clearance (Labarrere and Kassab, 2021)	Non-inflammatory clearance of apoptotic cells; enhances virus and bacteria phagocytosis (Labarrere and Kassab, 2021)	Since IgM NAbs enhance pulmonary alveolar late apoptotic cell clearance (Litvack and Palaniyar, 2010), intravenous administration of IgM NAbs will intensify antiviral protection and late apoptotic cell removal in the lungs by alveolar macrophages (Litvack and Palaniyar, 2010; Labarrere and Kassab, 2021). Cysteine supplementation will improve immunological functions by enhancing GSH levels (Dröge and Breitkreutz, 2000; Ghezzi, 2011)
Innate immune cells (monocytes, macrophages, dendritic cells)	Innate immune cells use pattern recognition receptors to phagocytize microorganisms and apoptotic/infected cells, produce cytokines and activate adaptive immune cells; they also promote phagocytosis, tissue repair, immunoregulation, antigen presentation, and cytokine production. Excessive cytokine production during cytokine storm in SARS-CoV-2 infection causes macrophage dysregulation, severe tissue damage and organ failure (Labarrere and Kassab, 2021)	Monocyte-derived tissue macrophages are normally involved in phagocytosis, clearance of apoptotic cells, immunoregulation and antigen presentation, and pattern recognition proteins like CRP, innate IgM and complement facilitate phagocytosis of infected apoptotic cells promoting tissue repair. Dendritic cells and macrophages are involved in linking innate and adaptative immunity against viral infections and participate in antigen presentation, cytokine production and immune cell recruitment (Labarrere and Kassab, 2021)	Hyperinflammation in severe COVID-19 infection, causes a dysregulated macrophage response, excessive cytokine production and tissue damage (Labarrere and Kassab, 2021). Dendritic cell dysfunction and dendritic cell depletion during SARS-CoV-2 infection are associated with lower Interferon I response and poorer prognosis. Dendritic cell changes contribute to COVID-19 pathogenesis and increased susceptibility to worst outcomes especially in the elderly (Cerqueira Borges et al., 2021; Labarrere and Kassab, 2021). GSH, enhanced by cysteine supplementation Nrf2 activation, is essential to reestablish innate and adaptive immune functions including T-lymphocyte proliferation, phagocytosis and antigen presentation by macrophages and dendritic cells (Dröge and Breitkreutz, 2000; Ghezzi, 2011)
B lymphocytes, plasma cells	Low affinity high valency IgM antibodies neutralize/remove virus and bacteria and lack of IgM neutralizing antibodies enhances inflammation (Labarrere and Kassab, 2021)	B-lymphocytes/plasma cells generate innate and adaptive IgM antibodies to neutralize/remove virus/bacteria (Labarrere and Kassab, 2021)	SARS-CoV-2 infection is characterized by an excessive inflammatory response associated with a cytokine storm and a prominent lymphopenia affecting CD4+ T cells, CD8+ T cells, B cells and natural killer cells. Both lymphopenia and the cytokine storm determine increased COVID-19 disease severity and enhanced mortality. Cysteine supplementation will improve immunological functions by enhancing GSH levels (Dröge and Breitkreutz, 2000; Ghezzi, 2011)
Helper (CD4) T cells, cytotoxic (CD8) T cells	T helper type 1 cells (Th1 cells; CD4+ T cells) regulate macrophage recruitment and activation enhancing defense against pathogens, and cytotoxic CD8+ T cells eliminate infection (Labarrere and Kassab, 2021)	Th2 cells mediate and maintain humoral (antibody-mediated) immune response against pathogens but unsuccessful control of the cytokine storm by Th2 cells in SARS-CoV-2 infection is associated with severe COVID-19 disease (Labarrere and Kassab, 2021)	SARS-CoV-2 infection is characterized by an excessive inflammatory response associated with a cytokine storm and a prominent lymphopenia affecting CD4+ T cells, CD8+ T cells, B cells and natural killer cells (Chen and Wherry, 2020; Labarrere and Kassab, 2021). Both lymphopenia and the cytokine storm determine increased COVID-19 disease severity and enhanced mortality (Labarrere and Kassab, 2021). Cysteine supplementation improves T-cell functions by enhancing GSH levels (Dröge and Breitkreutz, 2000; Ghezzi, 2011)

SARS-CoV-2, severe acute respiratory syndrome coronavirus 2; COVID-19, coronavirus disease 19; MyD88, Myeloid differentiation primary response 88; TRIF/TRAM, TRIF (TLR4 signaling)-related adapter molecule (TRAM).

## Author contributions

All authors listed have made a substantial, direct, and intellectual contribution to the work and approved it for publication.

## Conflict of interest

The authors declare that the research was conducted in the absence of any commercial or financial relationships that could be construed as a potential conflict of interest.

## Publisher’s note

All claims expressed in this article are solely those of the authors and do not necessarily represent those of their affiliated organizations, or those of the publisher, the editors and the reviewers. Any product that may be evaluated in this article, or claim that may be made by its manufacturer, is not guaranteed or endorsed by the publisher.

## Abbreviations

SARS-CoV-2: Severe acute respiratory syndrome coronavirus 2
COVID-19: coronavirus disease 19
ROS: reactive oxygen species
GSH: glutathione
Keap1: Kelch-like ECH-associated protein 1
Nrf2: Nuclear factor erythroid 2-related factor 2
ARE: antioxidant response element
GSSG: GSH disulfide
NADPH: nicotinamide adenine dinucleotide phosphate
NOX: NADPH oxidase
ARDS: Acute respiratory distress syndrome
NFκB: nuclear factor-κB
ACE2: angiotensin converting enzyme-2
NETs: neutrophil extracellular traps
rTEM: reverse trans-endothelial migration
NAC: N-acetylcysteine
CRP: C-reactive protein
HSPGs: heparan sulfate proteoglycans
GCS: glutamyl-cysteine synthetase
TMPRSS2: transmembrane protease serine 2
TLRs: Toll-like receptors
