# Supplementary material for: Glutathione deficiency in the pathogenesis of SARS-CoV-2 infection and its effects upon the host immune response in severe COVID-19 disease
Source: Front Microbiol. 2022 Oct 6;13:979719. doi: 10.3389/fmicb.2022.979719 (PMC9582773; doi:10.3389/fmicb.2022.979719)
Supplement: Supplementary file 1 [file Data_Sheet_1.PDF]

Table 1. Multi-weaponry approach involving glutathione (GSH) enhancers, nuclear factor erythroid 2 p45–related factor 2 (Nrf2) activators, toll-like receptor (TLR) inhibitors/immunomodulators, C-reactive protein (CRP) level reduction, natural and immune immunoglobulin M (IgM) enhancement and immune cell function recovery against SARS-CoV-2 infection and COVID-19 disease.

	**Proinflammatory effects**	**Anti-inflammatory effects**	**Treatment effects on SARS-CoV-2/COVID-19**
**GSH Enhancers** (N-acetylcysteine [NAC], glutamine, cysteine [cystine], glycine)	GSH is fundamental to sustain an adequate function of the immune system, particularly affecting the lymphocyte activity since low GSH levels inhibit T-cell proliferation and immune response (2,59-63). GSH levels in macrophages, directly affect the Th1/Th2 cytokine response (87). GSH is capable of scavenging ROS through Nrf2-mediated heme oxygenase-1 induction and enhancing M1-like macrophage polarization regulation, showing that GSH may be a useful strategy to increase the human defense system (91-93). GSH increases activation of cytotoxic T cells in vivo, and adequate functioning of T lymphocytes and other cells depends upon cellular supplies of cysteine (106-108)	GSH inhibits production of most inflammatory cytokines, and it is needed to keep an adequate interferon gamma production by dendritic cells, essential for intracellular pathogen host defense (60,67,88,89). The principal function of endogenous GSH is not to limit inflammation but to fine-tune the innate immune response to infection (14,69,74,90)	Administration of free radical scavengers could benefit the most vulnerable SARS-CoV-2-infected patients (46) Many antioxidants like *GSH, and NAC* inhibit viral replication (87). GSH precursors like NAC, glutamine, cysteine (cystine) and glycine, and Nrf2 inducers like sulforaphane can enhance GSH production and increase nuclear Nrf2 translocation and antioxidant response element (ARE) transcription (47,166,244-247). Since GSH inhibits viral replication and decreases IL-6 levels, liposomal GSH could benefit COVID-19 patients having SARS-CoV-2-induced cytokine storm and redox imbalance (102). NAC and GSH directly suppress spike protein receptor-binding domain-ACE2 binding functions of various SARS-CoV-2 variants (269)
**Nrf2 Activators** (Sulforaphane, melatonin)	Nrf2 activation suppresses ROS in antigen-presenting dendritic cells enhancing their capacity to interact with and promote the transformation of naïve CD8 T cells into cytotoxic T lymphocytes enabling cytotoxic T-cells to eliminate virally infected cells (61,116,249,253)	Nrf2 activation regulates antioxidant responses to modify cellular redox states from predominantly pro-oxidant to antioxidant, and, in an antioxidant environment, macrophage phenotypes shift from M1 pro-inflammatory to M2 anti-inflammatory, reducing the probability of cytokine storms, ARDS, and lethality (249,260,261)	Antioxidants (GSH, GSH enhancers) or Nrf2 inducers (sulforaphane, melatonin) are potential viable therapies for viral-induced diseases; Nrf2 activators like sulforaphane have a potential role with dual antiviral and anti-inflammatory properties in the management of COVID-19 pneumonia (242,252,253,259,261) and LONG COVID (264,265)

**Toll-like receptors (TLRs)**	TLR4 in the heart and lungs causing aberrant TLR4 signaling favors the proinflammatory MyD88-dependent (canonical) pathway instead of the alternative TRIF/TRAM-dependent anti-inflammatory and interferon pathway (218). TLR4 activation in platelets whether by pathogen- (viremia) or damage-associated molecular patterns induces a prothrombotic and proinflammatory state (220). Activation of endosomal TLR7/8 during SARS-CoV-2 may increase the inflammatory response resulting in severe and potentially lethal immunopathological effects in COVID-19 patients as consequence of the simultaneous release of pro-inflammatory cytokines and chemokines (304)	TLRs play a key role in microorganism and viral particle recognition and activation of the innate immune system and although pathogen-associated molecular pattern (PAMP) recognition by TLRs is crucial for host defense responses to pathogen infection, *aberrant activation of TLR signaling* by PAMPs, *mutations of TLR signaling molecules*, and *damage-associated molecular patterns (DAMPs)-mediated TLRs signaling activation* are responsible for the development of chronic inflammatory diseases (209-213)	GSH and GSH enhancers could neutralize oxidation radicals generated during TLR-mediated mitochondrial ROS production and directly affect SARS-CoV-2-mediated cellular and tissue damage (218); TLR inhibitors/immunomodulators could become promising treatments for severe COVID-19 (304,305)
**C-reactive protein**	*Pentameric native (n)CRP*-FcɣRI/FcɣRIIa: increases inflammatory cytokine release; nCRP- FcɣRIIb maintains a predominant anti-inflammatory effect; *non-native (nn)CRP* enhances inflammation and complement activation; induces atherogenesis; mostly proinflammatory; *monomeric (m)CRP* promotes chemotaxis; increases IL-8, MCP-1 and nitric oxide; induces ROS; mCRP-FcɣRIII induce inflammation; promotes adhesion molecule expression, thrombosis and atherogenesis (137)	*Pentameric nCRP* bound to phosphorylcholine (PC) or lysoPC-apoptotic cells, C1q and factor H: enhance phagocytosis; nCRP-FcɣRs: M2 response; *nnCRP* binds atherogenic LDL, reduces foam cell formation and could also be atheroprotective; *mCRP* is mainly proinflammatory and not anti-inflammatory (137)	Binding of CRP to SARS-CoV-2 virus and/or the cell membrane can impair subsequent virus attachment and entry into the cell (137). CRP apheresis could reduce CRP levels and inflammation (234,235), and reduced GSH, which has the anti-inflammation and anti-oxidation effects, can significantly decrease the plasma concentrations of CRP (233)

**Natural (innate) and immune (adaptive) immunoglobulin M (IgM)**	*Lack of innate and adaptive IgM* allows cell necrosis and inflammation and prevents apoptotic cell clearance (137)	Non-inflammatory clearance of apoptotic cells; enhances virus and bacteria phagocytosis (137)	Since IgM NAbs enhance pulmonary alveolar late apoptotic cell clearance (330), intravenous administration of IgM NAbs will intensify antiviral protection and late apoptotic cell removal in the lungs by alveolar macrophages (137,306). Cysteine supplementation will improve immunological functions by enhancing GSH levels (59,60)
**Innate immune cells (monocytes, macrophages, dendritic cells)**	Innate immune cells use pattern recognition receptors to phagocytize microorganisms and apoptotic/infected cells, produce cytokines and activate adaptive immune cells; they also promote phagocytosis, tissue repair, immunoregulation, antigen presentation, and cytokine production. Excessive cytokine production during cytokine storm in SARS-CoV-2 infection causes macrophage dysregulation, severe tissue damage and organ failure (137)	Monocyte-derived tissue macrophages are normally involved in phagocytosis, clearance of apoptotic cells, immunoregulation and antigen presentation, and pattern recognition proteins like CRP, innate IgM and complement facilitate phagocytosis of infected apoptotic cells promoting tissue repair. Dendritic cells and macrophages are involved in linking innate and adaptative immunity against viral infections and participate in antigen presentation, cytokine production and immune cell recruitment (137)	Hyperinflammation in severe COVID-19 infection, causes a dysregulated macrophage response, excessive cytokine production and tissue damage (137). Dendritic cell dysfunction and dendritic cell depletion during SARS-CoV-2 infection are associated with lower Interferon I response and poorer prognosis. Dendritic cell changes contribute to COVID-19 pathogenesis and increased susceptibility to worst outcomes especially in the elderly (137,307). GSH, enhanced by cysteine supplementation Nrf2 activation, is essential to reestablish innate and adaptive immune functions including T-lymphocyte proliferation, phagocytosis and antigen presentation by macrophages and dendritic cells (59,60)
**B lymphocytes, plasma cells**	Low affinity high valency IgM antibodies neutralize/remove virus and bacteria and lack of IgM neutralizing antibodies enhances inflammation (137)	B-lymphocytes/plasma cells generate innate and adaptive IgM antibodies to neutralize/remove virus/bacteria (137)	SARS-CoV-2 infection is characterized by an excessive inflammatory response associated with a cytokine storm and a prominent lymphopenia affecting CD4+ T cells, CD8+ T cells, B cells and natural killer cells. Both lymphopenia and the cytokine storm determine increased COVID-19 disease severity and enhanced mortality. Cysteine supplementation will improve immunological functions by enhancing GSH levels (59,60)

**Helper (CD4) T cells, cytotoxic (CD8) T cells**	T helper type 1 cells (Th1 cells; CD4+ T cells) regulate macrophage recruitment and activation enhancing defense against pathogens, and cytotoxic CD8+ T cells eliminate infection (137)	Th2 cells mediate and maintain humoral (antibody-mediated) immune response against pathogens but unsuccessful control of the cytokine storm by Th2 cells in SARS-CoV-2 infection is associated with severe COVID-19 disease (137)	SARS-CoV-2 infection is characterized by an excessive inflammatory response associated with a cytokine storm and a prominent lymphopenia affecting CD4+ T cells, CD8+ T cells, B cells and natural killer cells (137,308). Both lymphopenia and the cytokine storm determine increased COVID-19 disease severity and enhanced mortality (137). Cysteine supplementation improves T-cell functions by enhancing GSH levels (59,60)

Abbreviations: SARS-CoV-2: severe acute respiratory syndrome coronavirus 2; COVID-19: coronavirus disease 19; MyD88: Myeloid differentiation primary response 88; TRIF/TRAM: TRIF (TLR4 signaling)-related adapter molecule (TRAM).
